# Co-creating cultures of sustainability and co-imagining the teaching green building: the use of a participatory Photovoice process in a HPGB context

**DOI:** 10.1186/s42055-022-00047-y

**Published:** 2022-09-05

**Authors:** Kai Reimer-Watts, Esther Abel, Simon Coulombe, Manuel Riemer

**Affiliations:** 1 Department of Psychology, Wilfrid Laurier University, Waterloo, Canada; 2 Département des Relations Industrielles, Relief Research Chair in Mental Health, Self Management, and Work, Université Laval, Québec City, Canada; 3 VITAM Research Centre on Sustainable Health, Québec City, Canada

**Keywords:** Culture of sustainability, Climate change, Green buildings, Climate solutions, Sustainability, Action research, Culture, Empowerment, Engagement, Participation

## Abstract

Despite understanding the severity of the climate crisis, global action remains highly insufficient to address this challenge. Buildings are significant contributors to climate change due to their substantial global emissions, but can also contribute to urgent climate solutions. High-performance green buildings (HPGBs) can reduce operational building-level emissions dramatically, and potentially offer other benefits that support building users’ wellbeing and sustainable behaviours. HPGBs can provide useful environments to engage and influence culture and can act as publicly visible symbols of emergent local clean economies. However, a gap remains in knowing how best to support the emergence of citizen-led cultures of sustainability (COS) within green building spaces, an effort that could also help address the noted ‘performance gap’ of green buildings that has been linked to occupant behaviours. With the intention of investigating and supporting a growing citizen-led COS in a green building, this study applied an empowerment-based *Photovoice* method in the context of the HPGB evolv1, located in Waterloo, Ontario, Canada. Six building users (citizens) took and selected photographs, participated in facilitated group discussions and individual interviews, and contributed toward three public exhibitions based on research findings.

Based on thematic analysis, findings suggest building citizens know what a COS means to them, and existing barriers and enablers within/around the evolv1 building toward achieving this. In addition, participants recognized the impact of specific green building features on their own personal sustainability-related values and practices, including the influence of sustainability symbolism within the building environment. Lastly, participants articulated specific recommendations for further promoting and growing a COS at evolv1. Significant themes identified are discussed in relation to and expansion of Cole (2014)’s Teaching Green Building (TGB) Model for Learning, providing preliminary insights into the degree to which evolv1 may or may not presently embody key aspects of a TGB. This study contributes to deepening understandings of how researchers and building citizens can support the emergence of COS within green buildings and related environments, with key takeaways that can be usefully applied to other settings, and theoretical and practical implications. Research findings encourage action toward supporting engaged, citizen-led COS, to advance more sustainable futures.

**Supplementary Information:**

The online version contains supplementary material available at 10.1186/s42055-022-00047-y.

## Abstract (plain language summary)

There is not enough action to address the global challenge of climate change, despite understanding how severe this crisis is. While emissions from buildings do contribute significantly to climate change, buildings can also contribute to urgent climate solutions. High-performance green buildings (HPGBs) can reduce operational building-level emissions dramatically and offer other benefits, including potentially supporting the wellbeing and sustainable behaviours of building users. HPGBs can provide an important place to engage and influence culture toward supporting a culture of sustainability (COS), and can act as publicly visible symbols of the new clean economy. However, a gap remains in how best to support the emergence of cultures of sustainability led by building users within green building environments. To investigate and support an emergent citizen-led COS in a green building, this study applied an empowerment-based *Photovoice* method in the context of a HPGB, named evolv1 and located in Waterloo, Ontario, Canada. As part of this *Photovoice* study, six building users (citizens) took and selected photos, participated in facilitated group discussions and individual interviews, and contributed toward three public exhibitions based on research findings.

Thematic analysis of the data resulted in findings suggesting that building citizens know what a COS means to them, and existing barriers and enablers within the evolv1 building toward achieving this. In addition, participants recognized the impact of specific green building features on their own personal values and practices related to sustainability, including the influence of sustainability symbolism within the building environment. Participants also shared specific recommendations for further promoting and growing a COS at evolv1. Findings are discussed in relation to and expansion of Cole (2014)’s Teaching Green Building Model for Learning, providing early insights into the degree to which evolv1 may or may not presently embody key aspects of a Teaching Green Building. This study deepens understandings of how researchers and building citizens can support the emergence of cultures of sustainability within green buildings and related environments, with key takeaways that can be usefully applied to other settings, and both theoretical and practical implications. Research findings encourage action toward supporting engaged, citizen-led COS, for more sustainable futures.

**Supplementary Information:**

The online version contains supplementary material available at 10.1186/s42055-022-00047-y.

An increased understanding of the severity of the global climate crisis has not been matched with the required actions and societal changes necessary to mitigate this problem [1–3]. Despite the comprehensive scientific understanding we now have of the immense threat climate change poses to human society, the public spaces and infrastructure people move through in our cities, businesses and even places of learning often have little to no imagery or ‘action cues’ relating the critical importance of sustainability and individual and collective action in response to this crisis. Worse, most of these spaces are direct contributors to the problem, as the built environment is estimated to be responsible for roughly 40% of greenhouse gases (GHGs) worldwide [4]. Much research has shown that, far from being ‘passive’ infrastructure, the built environment can have an enormous influence on behaviours and wellbeing, including individual and collective willingness and opportunity to engage with sustainability [5–7]. Physical buildings perform utilitarian functions by providing places of work, home life and leisure, and also fulfill a clear symbolic function, reflecting many of the ideas, dominant values, aesthetics and ideologies of an age [8].

Buildings can be a critical part of the solution to climate change. The International Energy Agency has predicted that to have any chance at staying below a 2 °C global temperature rise – the stated upper limit of the 2015 Paris Climate Agreement – all building-related CO2 emissions must drop by 85% below 2018 levels by 2060 [9], and all new buildings must be zero carbon by 2030 latest [10]. High-performance green buildings (HPGBs) in particular offer enormous potential to address the technical challenge of reducing building emissions, to provide an important place to engage and influence culture, and to act as publicly visible symbols of the new clean economy [11]. Yet HPGBs also struggle with a clear ‘performance gap’ that has been linked to a lack of a culture of sustainability (COS, defined below) amongst building citizens, among other factors (e.g., [12, 13]). This gap refers to the difference between the projected operational energy and resource use of the building and actual use once people inhabit the building, which often falls short of anticipated higher levels of sustainability [12]. The difference has been linked in part to unsustainable human behaviour by building users, which can be addressed through supporting the development of a COS in building environments.

According to Dreyer et al. [14] a COS is characterized by “shared values, symbols, rituals, and practices grounded in sustainability principles leading to individual and societal choices that promote environmental protection, social justice, and well-being, and a supportive economy” (p. 5). Sustainability values, symbols, rituals, norms and practices must all be supported in some way for an effective COS to emerge and be sustained within a given environment [14, 15]. Ideally, these core attributes are mutually reinforcing in a positive feedback loop, for instance with shared values and norms leading to the creation of sustainability symbols, which may in turn reinforce or inspire more sustainable individual and group practices, rituals and behaviours. Such practices can then serve to further cement a belief in and commitment to a COS, creating a virtuous cycle (e.g., [16]). We define sustainability here as including a balance of environmental, economic and social considerations that can be sustained together over the long-term [17], importantly including considerations of fairness and social justice (e.g., [18]). A useful interpretation of sustainability that we draw from here is an approach “where wider questions of social needs and welfare, and economic opportunity, are integrally related to environmental limits imposed by supporting ecosystems” ([19], p. 78). For a more thorough discussion of COS and its relationships with sustainable values and behaviours, see for example [14], and [16].

Despite a clear emphasis on many key aspects of sustainability in the overall development and operation of HPGBs – albeit with some criticism of a common lack of focus on social equity in high-end green building spaces (e.g., [20]) – unsustainable use of otherwise mostly sustainable buildings and workplaces continues. This contributes to the green building performance gap and limits the potential of HPGBs as spaces to help ‘drive’ a much-needed sustainability transition across societies [21, 22]. This unsustainable use of HPGB spaces can be seen as – in part – a predictable byproduct of the broader cultures that continue to dominate modern societies today and condone many largely unsustainable practices as socially acceptable (e.g., leaving workplace lights on overnight in an unoccupied building, among many other examples). In contrast to a COS and alongside other factors that may also promote unsustainable behaviours, these socially accepted unsustainable practices could be said to both inform and be in part caused by the dominant ‘cultures of unsustainability’ [23] that many individuals already operate within. Such cultures may predictably influence individual and group behaviour within a given environment to be less sustainable, including in HPGBs. Developing more sustainable cultures and prefiguring desired changes takes intentional effort toward far deeper sustainability engagement from many people involved in a given building (or other) environment, to co-create and co-define a shared, emergent COS together (e.g., [16]). Importantly – and informing the guiding conceptual framework for the present study – engaging people in empowering processes that centre considerations of both individual/community wellbeing and environmental sustainability have been shown to be crucial to the development of co-creating a long-lasting COS (e.g., [14, 16]). The emphasis in environmental education on both “action competence” [24] and “emancipatory” learning approaches [25] can here align usefully with community psychology’s focus on individual and collective empowerment and wellness [26, 27] – as Harré et al. [16] argue, providing a useful pathway toward iterative sustainability engagement processes based in equity, listening, practice and reflection.

While the technology required to achieve HPGBs is already largely established, the social dimensions of how to support and co-create micro-cultures of sustainability within such spaces is, although a critical and emerging field, still far less so (for a theory of change in developing a COS in a HPGB context, developed in the context of the green building ‘evolv1’ which this study is also situated in, see [14]). Efforts to develop COS in green buildings are complex and have recently been met with both novel successes and challenges (see for example, [28], also situated in evolv1) with many implications for those interested in promoting such a culture in these unique contexts. This study aims to contribute to this emerging field, by investigating factors that promote a shared COS amongst building users (or ‘citizens’) through participatory co-creation of and critical reflection on this culture within the HPGB environment of the evolv1 building, located in Waterloo, Ontario, Canada, described below. This participatory action research project builds on the perspectives of a group of building citizens within evolv1, most of whom were already engaged with ongoing COS efforts in the building, drawing on their expertise in line with empowerment-oriented community engagement practices (e.g., [29, 30]) and as part of a broader COS-focused research project situated in the building. The modified *Photovoice* methodology described below was applied with participants in the spirit of action research (e.g., [31]), which is designed for the research to have a direct impact on change – in this case, towards further contributing to the development of a COS at evolv1, and potentially beyond.

## Building a COS: environmental education and ‘Teaching Green Buildings’

Recent research shows that a closer collaboration between the arts and social sciences may be key to developing the relevant messaging and symbolism required to support a strong COS (e.g., [32–34]). Yet despite the potential for green buildings to create a highly symbolic public statement as pioneers of sustainability and the transition to a clean economy, the role of symbolism, the arts and educational communication on sustainability have often been overlooked as potentially powerful features of many green building spaces [8]. Most green buildings today are not designed to explicitly *communicate* sustainability so much as they are designed to *operate* sustainably – meaning that for those interacting with the building it is still largely unclear “whether the message of the importance of sustainability is received” ([8], p. 827).

Given that each new green building represents a capital investment that is often in the hundreds of millions of dollars, and given the urgent nature of supporting sustainability practices across all of society to address the climate crisis, it is critical to find ways to leverage these green building spaces that many people already access to support deeper cultures of sustainability. To grow a COS within this context, green buildings must do more than simply operate sustainably; they must also engage users meaningfully on adopting and co-creating sustainable values, norms, symbolism and practices, increasing sustainable human behaviours. One way to do this is to aspire to create ‘Teaching Green Buildings’ [5], which this study aims to build on and support.

**Fig. 1:**
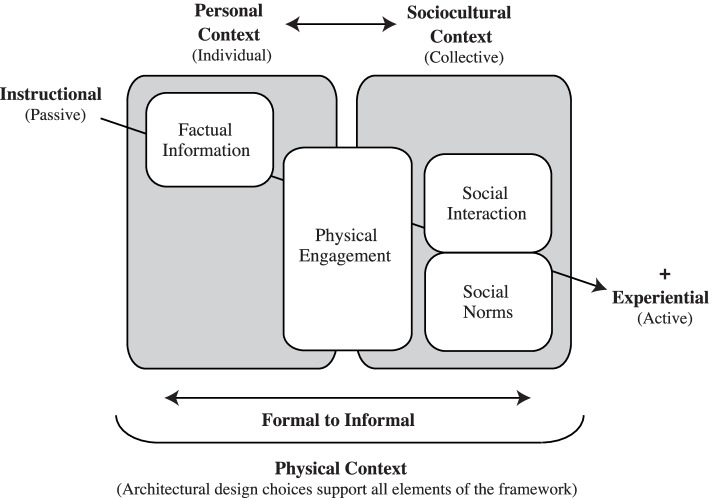
The Teaching Green Building Model for Learning. Reproduced with permission from Cole [5], further re-use of this figure is only allowed with permission of Cole [5]

Green school buildings are some of the early pioneers in the work of building a COS within green building spaces. These are buildings that are built to high sustainability standards as well as have a clear educational function and mandate that shape their use by their students, teachers, staff and the broader community to offer immersive environments for sustainability [5, 35]. The work of Cole & Altenburger [6], and that of Cole [36] both look at the use of multiple methods to support environmental education and engagement within a green building environment, building from the development of [5] ‘Teaching Green Building Model for Learning’ (p. 841; see Fig. 1 below, hereafter TGB Model), a conceptual framework that the current study builds on. This model is significant as it links environmental education and architecture to create what Cole [5, 36] has termed a ‘Teaching Green Building’ (TGB) in order to “engage its users in the environmental story of the building … [and] ideally achieve a delicate balance between delivering an efficient building and one that additionally serves as a meaningful “call to action” for occupants” ([36], p. 119).

While situated initially within a middle school context, the below model remains highly relevant for informing approaches to sustainability education and engagement within other green building contexts also, as explored further in Cole [36]. In this model, the horizontal axis represents a spectrum of engagement from formal to informal learning and individual to collective engagement, while the vertical axis illustrates the range of passive to active or experiential learning opportunities in the space. Notably, informal learning is shown to take place most within a sociocultural or collective context, through active engagement with peers and community, while the architectural design of the space ideally serves to support all elements of the framework.

According to Cole [5] each of the squares within the framework can be considered a ‘design pattern’ for shaping a TGB, providing further insight into *how* the physical context of a building can support formal to informal green learning opportunities, including through processes involving factual information, physical engagement, social interaction, and social norms around sustainability (see [5] for further details). Engagement here is identified “on a spectrum from person–environment interaction (personal context) to person–person interaction (sociocultural context), all supported by the physical environment (physical context)” (p. 841), resulting in a ‘web of possibilities’ for engagement with environmental issues in and around the building [5]. Relevant to this study, a green building’s architectural design can also embody “symbolic design choices, such as forms and materiality, [that] tell a nuanced story that can variously work for and against the overall environmental messaging” ([5], p. 849), underscoring the nuanced ways in which these buildings may communicate sustainability (or not) to those who use them.

In total, this model serves to emphasize the often underappreciated influence that the built environment can have on individual and collective experiences and learning processes, including of sustainability. It also suggests a useful pathway for how green buildings can do more than simply operate sustainably but can also play an active role in promoting a COS within and beyond their walls, in part by becoming TGBs. This is particularly important as green building features on their own, without intentional direct sustainability engagement and education, are insufficient to building a COS (e.g., [5, 12, 37, 38]). While being a TGB is not the only factor supporting a COS within green building spaces, as argued by Cole [36] a thoughtfully designed TGB space can be a significant contributor to this effort with positive environmental and social impacts, providing “exciting possibilities for making positive change through built form… from symbolic meaning to formal/informal environmental education to place-making and environmental behavior change” (p. 121). It is our view that for evolv1 to successfully support a COS within it, it must be shaped into becoming some kind of TGB, empowering building citizens and others to directly engage in and further sustainability.

To support the growth of a COS and core attributes of a TGB in evolv1, there is a clear need to engage those who regularly occupy the building in more direct, experiential, and active opportunities for sustainability engagement within this space. The *Photovoice* research method developed by Wang and Burris [39, 40] has demonstrated potential to be one such approach to engagement with people who use specific spaces to be able to actively engage and reflect on them and was adopted for this study. As a visual arts-based method (see [6]), it also meets previous calls for closer collaboration between the arts and social sciences as key to developing the relevant messaging and symbolism required to support a strong COS.

## *Photovoice* as a tool for empowerment toward growing a COS

*Photovoice* is a well-known participatory action research method for social change [41], and has been applied previously toward supporting the growth of a COS (e.g., [6]). The method combines the strengths of both visual and oral communication, by empowering participants to take and select photos of their environment and then use these photos as prompts for group and/or one-on-one discussions around specific topics of shared interest. *Photovoice* was developed as a tool that can be used by community members to help amplify their experiences to speak out on issues of shared concern, encouraging critical dialogue and reflection. With participants’ permission, these experiences can then also help influence decision-makers and a broader community toward specific actions [40].

*Photovoice* has also been identified as being particularly useful for grappling with complex or ‘wicked’ problems, including “environmental conditions and issues, which often lie at a nexus point of science and society” ([42], p. 53), and for its potential to catalyze engagement on sustainability at both an individual and community level. Building on previous COS work (e.g., [5, 14]), and reflections on the flexibility and adaptability of the *Photovoice* method to different community contexts and research interests (e.g., [43, 44]), the current study applied a modified *Photovoice* methodology as an empowering tool for engaging building citizens in developing a COS within the context of the evolv1 green building. To build a strong COS, there is a clear need to create links and relationships between how sustainability values and norms translate and/or relate to sustainability symbols and practices (if at all), and vice-versa. This research aimed to reveal and support these interconnections through the application of a modified participatory *Photovoice* process.

## Setting

This current study is part of a broader longitudinal case study exploring the development of a culture of sustainability within the energy-positive high-performance green building evolv1, including considerations of environmental, personal and social wellbeing and their rich intersections in this context. This 5-year study started in 2018 and includes a comprehensive culture of sustainability strategy coordinated by a Manager of COS. Prior to participating in the study, most of the participants of this *Photovoice* project had been citizens of evolv1 long enough to have been engaged in some activities related to the COS programming in the building. Hence, this study contributes to the broader, ongoing COS research and action taking place at evolv1, that many building citizens have engaged with and contributed to. The study received a multi-institutional research ethics approval.

evolv1 is a 104,000 square foot, three story HPGB located in the David Johnston Research + Technology Park in Waterloo, Ontario, that was inspired by the local non-profit Sustainable Waterloo Region (SWR) and is owned and developed by The Cora Group [45, 46]. It is Canada’s first net-positive energy commercial multi-tenant office building, opened in November 2018, and as of March 2020 home to seven tenant organizations in diverse sectors including information technology, professional services, artificial intelligence, clean technology, startup mentorship, education, and non-profit – several of which are housed within a unique clean economy innovation hub called evolvGREEN that is focused on sustainability [45, 47]. Notably, while the building context is multi-tenant, as of the time of the study there had been no tenant turnover to date in the building. In addition, despite significant differences between the business sectors and practices of the seven tenant organizations, a rich, emergent COS supported through strategic COS programming and citizen-led efforts had already begun to develop in the building prior to the start of this study, connecting these diverse organizations and many of the people within them (for more on this, see [14, 28]). This made evolv1 an ideal place to study the development of a COS in a HPGB context.

To achieve being net-positive energy, evolv1 produces more clean energy than it consumes, due largely to the extensive solar panels that cover both its roof and parking lot – the building’s total clean technologies are expected to produce 108% of the projected building energy needs on site [46]. The building’s other major sustainable features include a geothermal well that recycles groundwater for heating and cooling (hence not depleting underground aquifers); a 40,000 liter cistern for collecting rainwater; 28 electric vehicle charging stations; a passive SolarWall® that preheats incoming fresh air; triple-glazed windows with daylight harvesting; a state-of-the-art HVAC system; and a 40-ft living wall, among others [45]. Lastly, evolv1 was constructed according to LEED Platinum principles, and is also the first office building to receive the Zero Carbon Building-Design Certification from the Canada Green Building Council [46].

evolv1 is also more than just a building, and is “meant to motivate, inspire, and educate the public about sustainable design” [48]. At the time of this study evolv1 also included a dedicated Manager of Culture of Sustainability – a unique position to help inspire this shared culture in the building and importantly, help connect all evolv1’s diverse tenants together in a common cause for sustainability, managed by SWR and supported by funds from the evolv1 research project [45]. Further, ongoing COS initiatives in evolv1 supported building-wide – including by all tenants and by building management – have strengthened the social connectivity between diverse tenants and evolv1 citizens through engaging together in common cause, including through a series of COS workshops; presentations; organizational-level dialogues, focus groups and initiatives on sustainability; emergent citizen-led sustainability groups; and ongoing building tours engaging building citizens, among other initiatives (see pp. 70–73 in [45]). This has enabled this *Photovoice* study to be well-supported within this broader context of growing a building-wide COS. Note that at the time of data collection, the first tenants had arrived in the building following its official opening 16 months prior.

## Objectives

The objectives of the *Photovoice* study were (1) to examine how participants are influenced by evolv1 as a HPGB, including the influence of evolv1’s building features on participants’ own sustainable values and practices; (2) to explore participants’ understanding of a COS, including their views on what could be done to further promote sustainable values and practices at evolv1; and (3) to identify how the evolv1 building communicates to participants through symbolism, including how participants understand and respond to perceived symbols in the building environment. There was also a fourth objective that is more exploratory: to explore to what degree evolv1 may or may not currently embody core aspects of a TGB, based on an analysis of participants’ perspectives and framed by the TGB Model (with no associated question). Supporting these objectives were four research questions:

- (Q1) What does a COS mean for citizens of the building, and what can influence its development?
- (Q2) What, if any, building features positively or negatively influence the sustainable values and practices of citizens and their organizations?
- (Q3) How does the building symbolically communicate to people and how do symbols in the building environment translate into citizens’ own sustainability-related values and practices?
- (Q4) What could be done to further promote sustainable values and practices at evolv1?

Lastly, a final objective of the study was to support the bottom-up, citizen-led development of a COS at evolv1, contributing to the broader ongoing COS research project taking place in the building of which this study was a part.

## Methods

### Participants

#### Recruitment

Participants were recruited via digital communication shared by all evolv1 tenant organizations with their members, as well as physical flyers placed throughout evolv1. To qualify to participate, applicants had to (a) be working in evolv1 with a tenant organization for at least 10 hours a week, and (b) be available for a minimum three 1-hour sessions, over a period of 3 weeks. A total of six applicants applied to participate in the study, all of whom met the above criteria and were accepted, forming a similar group size to previous *Photovoice* studies (for similar *Photovoice* sample sizes, see for example [49–51]; for variability of *Photovoice* sample sizes, see [43, 52]). All participants signed a written consent form and engaged in the research study voluntarily, with no direct monetary compensation, however, participants did receive a $10 gift card to a local café for each session attended.

The six study participants worked within four different tenant organizations in evolv1 (see *Demographics*, below). A mutually agreed-upon time to meet for the weekly 1-hour sessions with participants was established of 12-1 pm EST on Tuesdays, starting March 24, 2020, which was held weekly for four group sessions. In addition to the group sessions, all six participants opted to participate in one-on-one individual interviews of between 1 and 1.5 hours each (details below). The time spent with participants was derived based on common practice in comparable *Photovoice* research studies (e.g., [51, 53]).

#### Demographics

As of the start of the study, four of the study participants were between 22 to 32 years old, one participant was 42 to 52 years old, and one participant was more than 52 years old. Four participants self-identified as women, and two as men. Half the participants indicated having worked at evolv1 for less than 1 year, and half for between 1 to 2 years (notably, this time period is comparable to the length of time evolv1 had been open to public access as the building opened in November 2018). Hence, some participants had been working in the building since its official opening and most had participated in COS programming in the building of various kinds prior to engaging in the *Photovoice* study. Related to their highest level of education achieved, half the participants indicated completion of college or university, and half indicated that they had either completed some or fully completed graduate studies. All six participants self-identified as non-Hispanic white, or euro-American. Five of the participants identified their role in their organization as employee, and one participant identified their role as team lead within the organization. Lastly, four of the six participants worked for two different sustainability-focused organizations in the evolv1 building – though notably, the results of these participants were not substantially qualitatively different from those working for  the remaining two non-sustainability focused organizations.

### Procedure

#### Weekly group sessions

In line with previous *Photovoice* studies (e.g., [29, 54]), the study was structured as three mandatory 1-hour weekly group sessions and one optional final session over a one-month period to accommodate participants’ schedules, along with an optional individual interview for each participant. The first session took place in an evolv1 meeting room, transitioning to virtual sessions via Zoom video conferencing in session two following the onset of the COVID-19 pandemic. Sessions were spaced out with 1 week between each to provide sufficient time for participants to take and/or select photos in response to the specific theme(s) discussed that week (see the subsection below *A Note Regarding Research Adjustments to the COVID-19 Pandemic*, explaining how the process was adapted to the pandemic context). Relating to the core research questions, group sessions focused on the following content and themes: Week 1) Introduction to the *Photovoice* method, research topics, ethics, participants’ conceptualizations of sustainability and wellbeing, photography techniques, guidelines, and process forward; Week 2) Photos and discussion centering on participants’ experiences of wellbeing in the evolv1 building; Week 3) Photos and discussion centering on participants’ experiences of sustainability in the evolv1 building; Week 4) The ‘Future of Sustainability’, exploring participants’ recommendations of what should be done to increase wellbeing and sustainability at evolv1 (attended by five participants).

Sessions were facilitated by two doctorate-level researchers, the first and second authors, along with their research supervisor for the project Dr. Simon Coulombe. While the first author’s research focus was on evolv1’s influence on participants’ experiences of sustainability, the other researcher investigated research questions on wellbeing (see [55], in preparation). Starting in the second session, each session incorporated group dialogue around the photos taken or selected during the previous week, connecting this dialogue to that week’s theme and any reflections participants wished to bring forward (note that both researchers analyzed data from all four group sessions, as relevant to their focus area). Participants were oriented during all group sessions to particularly reflect on their experiences in the evolv1 building prior to the onset of the COVID-19 global pandemic, at which point all participants were required to work from home (see *A Note Regarding Research Adjustments to the COVID-19 Pandemic*, below). Notably, at this point at the very start of the pandemic, both the participants and the researchers fully expected to be returning to working in the evolv1 building soon. While that did not eventually happen as the COVID-19 restrictions ended up being maintained for several months, this period of uncertainty did provide a unique opportunity for participants to reflect on both their previous in-person experiences at evolv1, and to contrast experiences working from home and at the office, to identify what they may want to see changed or maintained at evolv1 upon their return in line with the themes of each weekly group discussion.

Aligned with common practice for *Photovoice* studies, in addition to question prompts prepared by the researchers related to the specific week’s theme, the short-hand mnemonic SHOWED approach was used as a way of focusing participants on the experiences and stories represented in the photographs and promoting critical reflective group dialogue (e.g., see [40, 51, 56]):

***﻿S*** *What do you* ***see*** *here?*

***H*** *What is really* ***happening*** *here?*

***O*** *How does this relate to* ***our*** *lives?*

***W*** ***Why*** *does this problem or strength exist?*

***E*** *How can we become* ***empowered*** *about this issue?*

***D*** *What can we* ***do*** *about it?*

#### Training and understanding sustainability

In addition to the areas of training described under *Procedure* above, the first session also included participants’ discussion of the meaning of ‘sustainability’ from their own perspectives, forming a rich group foundation for understanding sustainability informed by participants’ diverse perspectives. To help encourage some common ground of understanding and position participants’ understandings within a broader context, researchers also shared several brief and diverse conceptualizations of sustainability from relevant academic literature (e.g., [19, 57, 58]) – however, it was participants’ own understandings that formed the basis for all future discussions, photo-taking and photo-sharing related to sustainability. During the final individual interviews, all six participants were again asked to share what sustainability means to them, discussed further in the *Results and Discussion* section below.

#### Taking, selecting and sharing photographs

For each week following the first session introduction, participants were invited to take or select between 5 and 10 photos of spaces and ‘moments’ within and around evolv1 (including, if they felt it connected to evolv1, of their current work environment) that from their perspective in some way connected to that week’s theme. From these, participants were invited to share their top 2–3 photos at the following session, and present to the group on why they felt these specific photos represented the week’s theme (described further below). Establishing a limit on photos taken and shared helped ensure a predictable and usable number of photos for researchers (e.g., [59]). Given this limit, participants were also encouraged to carefully consider what they would like to photograph or select and why in advance of taking, selecting and submitting any photos. Photos were submitted weekly the day prior to the group session via a secure online portal, and participants were invited to submit short written captions and titles (if desired) to accompany each photo submission (though notably were not able to see each other’s photo selections and writing within the submission portal). Photos submitted included physical building features and spaces, evolv1’s surrounding environment, and other images including beyond evolv1 that seemed relevant to participants in suggesting sustainability and/or wellbeing in relating to that week’s theme.

Group sessions included a collective discussion on the specific photos selected by participants for that week, led by participants with infrequent prompting questions as appropriate asked by the facilitators. To support group discussion, facilitators also shared each photo virtually as it was discussed for all to see. Each participant both introduced their own selected photo(s) for that week and why they had selected them, and were encouraged to respond to other selected photos as well with further reflections and prompting questions. In total, these approaches resulted in rich, layered and constructive dialogues around the selected photos in each session, linking to that week’s theme.

Following each session, the three facilitators discussed amongst themselves what they felt may be common themes emerging from the data, and then presented these observations for the group’s feedback at the start of the next session, prompting further feedback from participants and refinement of proposed themes. These dialogues were open and re-shaped how researchers understood the themes emerging based on participant feedback, providing a useful starting place for further theme identification and refinement that took place during the data analysis process.

#### A note regarding research adjustments to the COVID-19 pandemic

While in a typical *Photovoice* study all photographs would be taken by participants themselves, this study was unique in that not all photos were taken by participants, with an additional comprehensive library of photos being taken by the researchers. This approach provided participants with the option of either submitting their own photos and/or selecting from the photo library created when deciding which photos to share for each week’s session (note that over the course of the study, all participants shared a combination of both some of their own photos, and some selected photos from this created library). Importantly, this approach is in line with the ‘flexibility advantage’ of *Photovoice*, which can be usefully adjusted to the realities of different community settings while still retaining its core elements (e.g., see [44]), and is often applied in a modified version from how Wang and Burris [40] originally conceptualized it (see Lal et al.’s [60] scoping review of *Photovoice* studies published between 1998 and 2011, most of which involved modified applications). For a similar study where participants were offered two different options of either taking their own photos or using select photos taken by others, see [61].

In the present study, the modification of the traditional *Photovoice* approach was made necessary due to significant restrictions to accessing the evolv1 building during the COVID-19 pandemic beginning in March 2020, as physical distancing measures and work-from-home orders were put in place both by the province of Ontario, evolv1 tenant organizations and the researchers’ university, requiring the research team to pivot from in-person to virtual meetings with participants starting in Week 2 of the study. As participants were suddenly required to work from home, many could no longer access evolv1 to take photos for future group sessions. Adapting the *Photovoice* method to suit this new reality, researchers secured permission to take several hundred photos documenting all publicly accessible/visible aspects of the evolv1 building’s interior and exterior in a single photo shoot between Week 1 and Week 2 of the study, creating a large virtual library of photos of evolv1 (*n* = 207) that participants had access to and could select from for future sessions. Efforts were made to create a comprehensive photo library of evolv1 with minimal researcher bias, including photos both of building features that could be categorized as being clearly sustainable (e.g., the solar PV panels), and other features with less or no evident link to sustainability. Hence, the photo library aimed to be a comprehensive documentation of evolv1’s publicly accessible features without being biased towards profiling any particular features. Participants then retained the agency of selecting which photos from this library they felt were most relevant to the theme of that week’s session and/or taking their own photos, including with the option to take photos of their current work environment outside of evolv1 if they felt aspects of this environment reminded them of something from their experience at evolv1. Participants then were asked to share 2–3 photos out of those they had selected and/or taken for each group session.

As a novel adaptation to standard *Photovoice* practice, this approach did succeed in enabling participants to reflect on the evolv1 space using selected or taken photos as prompts, despite not always being able to be physically present in the building to take their own photos (though many photos were taken by participants also, in addition to the researcher-created photo library). Hence, for the purposes of this study participants are described as either ‘taking or selecting photos’, and the adapted modified *Photovoice* method applied is described as a ‘hybrid’ physical-virtual approach (see *Limitations* for more information).

#### Individual interviews

Following the four group sessions, each participant was invited to participate in an optional one-on-one semi-structured interview led by one of the researchers to discuss their selected photos in greater depth, including how the photos linked to core research questions, as the final stage for participants in the research process (following other similar *Photovoice* studies, e.g., [54, 62]). All six participants agreed to participate in the interviews, which took between 1 and 1.5 hours per interview. An interview guide was created in advance by the researchers to guide the interview sessions. Sustainability-related questions included, *“What would you say a strong culture of sustainability in a green building like evolv1 might look like?”*, and *“Do you think the features that you’ve identified in the pictures as communicating messages about sustainability are actually impacting any of the values, norms and practices (i.e., actions) of people in the building?*”, among others.

#### Data recording, treatment and analysis

Following standard practice, all group sessions and interviews were audio-recorded and transcribed as part of the core data collected, along with collecting digital copies of all photos taken or selected (see [59]). Transcribed data was then de-identified and imported into the qualitative data analysis software *NVivo* for further analysis. Total data collected included participants’ submitted photos; photo captions; transcriptions of group sessions; and transcriptions of individual interviews.

Data analyzed included both the transcribed group sessions and individual interviews, using inductive coding consistent with standard practice for *Photovoice* projects (see [42, 63]) and a reflexive thematic analysis to identify overarching themes [64, 65]. Note that visual data within the photos themselves was not analyzed, both as analyzing visual data is an optional consideration for *Photovoice* projects, and as most photos were taken by the researchers, not participants (described above). However, photos selected by participants did form the basis for conversation in all group sessions and interviews, informing the discussions that were analyzed for the current paper, and were included in three final public exhibits based on the study (described below).

In line with standard practice (e.g., [43, 66, 67]) initial inductive coding used low inference codes to privilege participant language used and their own perspectives. These codes were then merged into categories resulting in a codebook with 32 overarching categories (grandparent codes) that each speaks to multiple themes, applying a thematic analysis. These grandparent codes were in turn grouped into sixteen larger meta-themes identified first by the first author and then confirmed and refined with input from both the remaining study facilitators (two of the other co-authors) and with participants, defining the overarching themes of the study. All three facilitators kept detailed notes of their reflections on the overall research and data analysis processes, returning to participants several times over the course of the study to confirm and further clarify research findings. Final themes that emerged were selected by the researchers based both on overall salience and relevance to answering the research questions and on a general consensus amongst participants that the theme was accurate and important to include. To maximize relevance, themes were carefully identified in response to the research questions, while ensuring they remained valid and representative of the data (e.g., [68, 69]). While the majority of the themes drew from similar statements and perspectives observed in the data shared by most participants, several themes were still included that drew from perspectives shared by only a few participants as they were deemed to be highly salient to answering the research questions, in line with the recognition that “even single codes may be important and transferable to other settings” ([70], p. 123). However, note that there were no themes that emerged based only on one participant’s perspectives – all themes represented the perspectives of at least two or more participants. Finally, the proposed final themes were all confirmed with participants via a follow-up survey (below), as a form of member checking (e.g., [71]).

#### Finalizing the themes

In addition to discussing and clarifying the evolution of core themes as they emerged over the group discussions, final themes were confirmed with participants via an online follow-up survey used to create the *Photovoice* exhibit (the same survey mentioned previously), providing the opportunity for participant feedback that was then incorporated to further refine understanding and presentation of the themes. This survey also provided participants with between 3 and 4 photo options to choose from per theme identified in the thematic analysis, enabling participants to then select in ranked order which photos they felt were most representative for each theme (ranked 1–4), as a means of informing which photos would be included in the public *Photovoice* exhibitions (described below).

The follow-up survey was designed on an online survey platform page, then shared via email with all participants. Proposed themes were presented in the survey in a similar format to that seen in Table 1 (p.11), below, with Research Question 1 (Q1) proposed themes presented first for participants to consider, respond to, and suggest potential accompanying photos for; followed by (once completed) Research Question 2 (Q2) proposed themes and potential accompanying photos; followed by Q3 and Q4 proposed themes and photos, all in the same format. At the end of each section of proposed themes, participants were asked to share their thoughts on the themes identified in relation to the research question (Q1 – Q4), reiterating that question. In addition, participants were asked here: if they were in agreement that the themes proposed were important and if not, then what should be modified; whether they felt there were any missing themes; and whether they had any other suggestions or comments based on the photo selections they’d proposed.

**Table 1:** Themes that emerged from the data, organized by research question

**(Q1) What does a culture of sustainability mean for citizens of the building, and what can influence its development?**	
***Q1 Themes*** (Q1-T1) Individual interest and commitment to sustainability (Q1-T2) Community-building for collective action on sustainability with shared purposes (Q1-T3) An empowering, healthy and enabling environmental context	
**(Q2) What, if any, building features positively or negatively influence the sustainable values and practices of citizens and their organizations?**	
***Q2 Themes*** (Q2-T1) Many evolv1 features already promote sustainable values, norms and practices (Q2-T2) Sustainability is not always ‘pure’ (Q2-T3) Some evolv1 features are actively discouraging more sustainable values, norms and practices (Q2-T4) evolv1 still embodies several ‘missed opportunities’	
**(Q3) How does the building symbolically communicate to people and how do symbols in the building environment translate into citizens’ own sustainability-related values and practices?**	
***Q3 Themes*** (Q3-T1) Certain building features clearly function as symbolic ‘green features’ (Q3-T2) Symbolic communication often requires ‘standing out’ (Q3-T3) What is missing or invisible in an environment can unintentionally create a ‘negative symbol’ for sustainability (Q3-T4) Sustainability communication and education are distinct from but connected to sustainability symbolism (Q3-T5) Concern over symbolic representation of sustainability, versus actual sustainability	
**(Q4) What could be done to further promote sustainable values and practices at evolv1?**	
***Q4 Themes*** (Q4-T1) Reconsider the function of spaces within and around evolv1 to center sustainability and community-building (Q4-T2) Combine existing symbolic communication with direct sustainability education and engagement (Q4-T3) Encourage more sustainable behaviours and discourage less sustainable behaviours (Q4-T4) Increase opportunities for social connection, nature connection, community-building and sustainability leadership	

Specific descriptions for each theme are defined further in Additional file 2: Appendix B

All participants completed the follow-up survey, with only minor feedback provided at this stage, primarily on which photos should be chosen to accompany which themes for the exhibits. With no stated disagreement and in fact many expressions of agreement on the themes proposed, the survey suggested significant consensus by participants on the final proposed themes. Only one suggestion for an additional theme was proposed by one participant, who proposed adding a theme that would be evolv1-specific and focus on the building’s alignment with sustainability – however, as such a theme would not have clearly answered any of the research questions or been easily transferable to other contexts beyond evolv1, and as it was suggested by only one participant, it was in the end not included in the final themes. The survey was completed by all six participants, and analysis of survey data informed the final exhibits.

#### Selection and final exhibits

As a form of action research, many *Photovoice* studies are accompanied by a public exhibit(s) sharing core research results to increase the social and policy impacts of the research, with participants’ consent (see for example [51, 54], among others). *Photovoice* exhibits are a powerful form of knowledge mobilization and translation of core research findings into the public sphere, to better influence and engage power holders and policymakers around issues of interest that emerged over the study (e.g., see [53]). Similarly, participants in this study were offered the optional opportunity to exhibit select photos along with descriptions of research themes that emerged and participants’ own de-identified reflections via written quotes in a public exhibit, which all participants agreed to. Two physical exhibits took place, the first at a local arts institute ‘THEMUSEUM’ in downtown Kitchener, Ontario, in August 2021, and the second at the evolv1 building itself in December 2021 and continued into late-2022. An online exhibit was also hosted on the website of local non-profit and evolv1 tenant Sustainable Waterloo Region and had a public launch event that took place on October 28, 2021, attended by over 100 people – including citizens of evolv1, local regional staff and policymakers, and members of the broader local community. The online exhibit can be accessed at www.sustainablewaterlooregion.ca/photovoice.

To inform the final content of the exhibits (all identical in content), in the survey, participants were asked to rank each grouping of 3–4 photos in order of most representative to least representative of the matching theme in question. Survey photo options provided were selected by researchers from participants’ prior photo selections relating to the theme in question. The survey also provided participants the opportunity to comment on why they ranked photo groupings in a particular way. Results of the survey were analyzed to inform the final photos included in the exhibits, resulting in 1–2 unique photos selected to represent each theme (for viewer interest, no photo was used for more than one theme). Once a photo was selected, quotes related to that photo and/or theme were identified from the data by the researchers and confirmed for use with participants. Each participant had the opportunity to review and modify all quotes using their language prior to public use. Several participants also joined in an optional follow-up meeting to the study led by the researchers to co-plan the exhibit in April 2021.

**Fig. 2:**
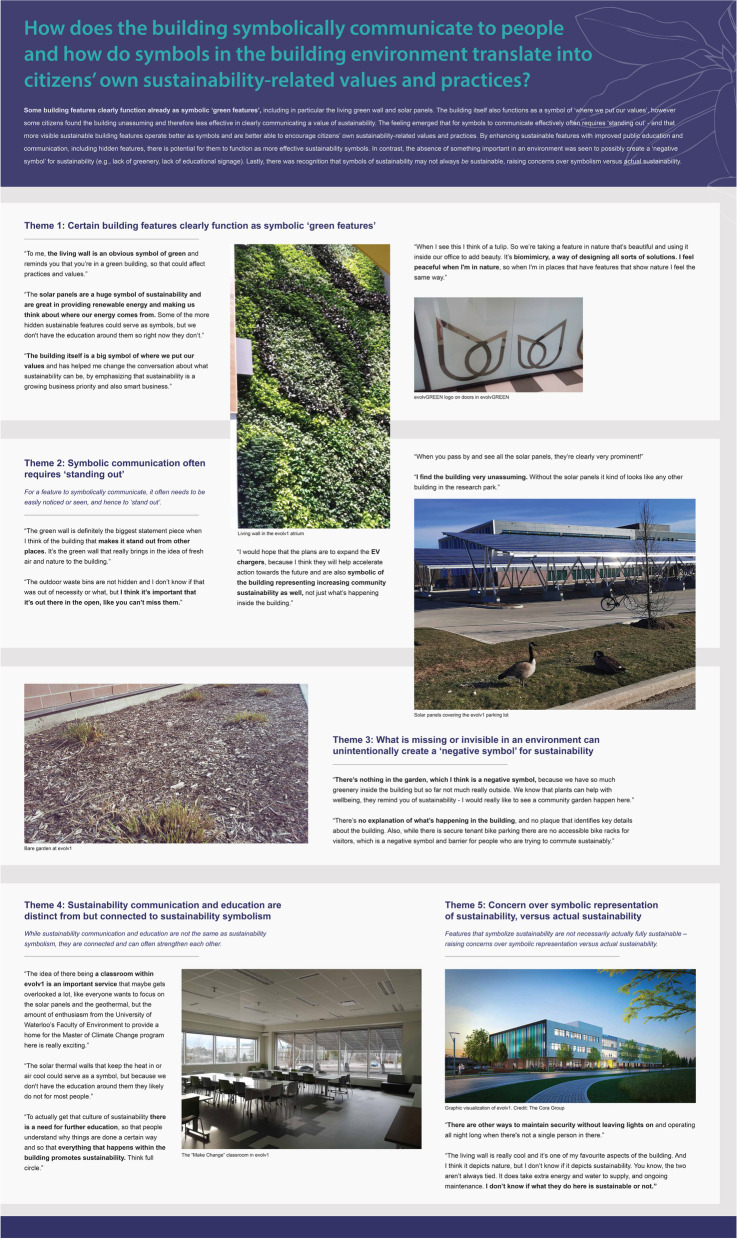
Panel in Response to Q3, Shared During the Photovoice Exhibits. Dimensions approximately 3.5′ × 5.5′. Further re-use of this figure is only allowed with permission from the authors

**Fig. 3:**
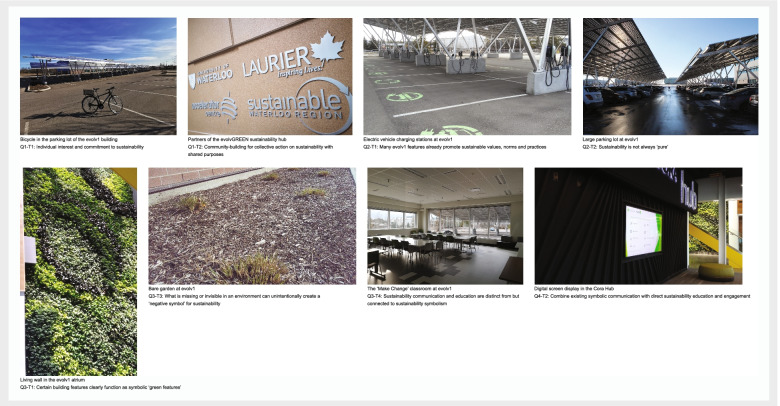
Examples of Significant Exhibit Photos. For more photos see Additional file 1: Appendix A, or www.sustainablewaterlooregion.ca/photovoice. Further re-use of this figure is only allowed with permission from the authors

In the end, 19 distinct photos were showcased representing sustainability in line with the sixteen themes presented, with each photo accompanied by one or more related quotes from participants. Selected photos and themes were matched together and arranged on four sustainability-related panels (see Fig. 2 for an example; there were also separate wellbeing-related panels, see [55], in preparation), printed and displayed physically in THEMUSEUM space, evolv1 building space, and displayed virtually in the online exhibit, along with a single introductory panel for the exhibit (see Fig. 3 for examples of pictures in the online exhibit).

## Results and discussion

Sixteen main themes emerged from the group discussions and one-on-one interviews in relation to the four research questions explored in the study (Q1-Q4), with participant feedback used to further refine the themes (discussed above). The subsections below present the results from participants’ understandings of a COS (Q1); their perceptions of how the evolv1 building influences their own sustainability values and practices (Q2) and how the building symbolically communicates (Q3); and their views on what could be done to further promote sustainable values and practices at evolv1 (Q4). These results represent sixteen qualitative themes shown in Table 1 (p. 11); within these identified themes, an analysis of the data revealed nuances as to what each theme meant to participants, discussed further in the form of key takeaways linked to previous research. These themes are framed further, where appropriate, in relation to the proposed TGB Model design patterns, to better assess in what ways evolv1 may or may not embody core aspects of a TGB based on the current study.

For increased clarity and presentation, results are presented within four distinct high level subsections below, each corresponding to one of the research questions. Each of these is then followed by a general discussion that further positions participants’ understandings and the study themes within a broader context of COS literature and lessons from the TGB Model, accompanied by the authors’ own views on important takeaways (labelled *Key Takeaway and Discussion*, below each results subsection).

### Growing a culture of sustainability

In response to Q1, *What does a culture of sustainability mean for citizens of the building, and what can influence its development?*, participants shared their perceptions of core characteristics of a COS that can be seen to exist along a broad continuum, and current enablers and barriers to growing such a culture in the context of evolv1. Participants made clear that what a COS means to them cannot be separated from their ability to actually enact it within evolv1, and shared their perceptions of to what degree a COS already exists in evolv1.

Descriptions of what a COS means to participants were linked to their own diverse understandings of sustainability, explored during the final individual interviews. Common understandings shared between multiple participants included understanding sustainability as being grounded in relationship between the self, society and a broader environmental context, articulated by one participant as having *“synergy with a connected world”* (P5). Another common description included balancing both environmental, economic and social aspects of a COS, including concerns for wellbeing – reflective of the ‘tri-factor’ of sustainability [17]. Notably missing from most (though not all) participant responses was a more critical reflection on the role of social justice in particular as a key component of sustainability – despite this connection being well-established for both sustainability (e.g., [18, 19]), and wellbeing (e.g., [26]).

COS was described by participants not as ‘one thing’, but as being comprised of multiple layered elements that can together co-create a more lasting sustainable culture. At the simplest level, participants identified the need for some degree of interest and commitment to sustainability by individuals who make up a culture (Q1-T1), demonstrated through individual sustainable actions (such as individuals biking or taking public transit to evolv1, or participating in sustainability initiatives, see Additional file 1: Appendix A, Figure A1). In one participant’s words: *“There should be an appetite to learn more about sustainability, thinking ‘how can I translate some of these things that I learn from this building into other aspects of my life?’”* (P4).

Participants also recognized that while individuals do have their own agency to take sustainability action, this agency is shaped and constrained by larger environmental and social contexts within which they are embedded. Hence, a COS was also seen to be shaped by intentional community-building for collective action on sustainability with shared purposes (Q1-T2), including the sense of ‘coming together’ through collaboration between multiple people and organizations in service of sustainability, creating a supportive social context (e.g., part of the focus of the evolvGREEN sustainability hub in evolv1). In participants’ words: *“We work better when we’re all together on something like this”* (P3), and *“If only one person is trying to be sustainable, it’s not going to have a lot of impact. It’s only impactful when everybody is doing it”* (P6).

Lastly, participants recognized the need for a healthy, supportive environmental context for people to connect in to help enable a COS to emerge (Q1-T3). This included having dedicated spaces to intentionally socialize with other building citizens and/or community members beyond one’s own organization, around particular areas of sustainability interest. One participant shared, *“I’d like to see the Hub [in evolv1] being used as a shared space where the tenant organizations can connect”* (P2). Participants emphasized how evolv1 itself, tenant organizations, and building and organizational management (among others) can all play a role in empowering building citizens to collaborate in intentionally envisioning and pursuing sustainability actions. *“People need to feel empowered to act with a clear understanding of how they can spark change”* (P4). Participants also recognized that acting on sustainability is closely tied to experiences of wellbeing within evolv1, and that current policies and cultural norms of tenant organizations and building management can have a significant effect on building citizens’ sustainability engagement. For example, one participant expressed frustration that, when bringing up the issue of soap dispensing continuously in the bathrooms with building management, *“they say they’d rather waste soap than have people frustrated that they can’t get soap”* (P2), clearly limiting citizens’ ability to take action on this issue. In contrast, another participant expressed appreciation that their organization’s sustainability norms *“makes you more cognizant of your actions”* (P1). Lastly, there was recognition of the broader environmental context in which evolv1 is situated, including the city of Waterloo and Waterloo region. Certain city features were seen to promote and help enable sustainable behaviours, including the nearby cycling and nature trails, and a light rail transit stop next to evolv1. However, the greater time required for biking or taking public transit when compared to driving, and the limited transit service to certain areas, were seen to be realities hindering more sustainable behaviours.

Participants also acknowledged the range of existing efforts to help build a COS in evolv1, many of which were ongoing at the time of the study with leadership from building tenants and the Manager of Culture of Sustainability. Unfortunately, the onset of the COVID-19 pandemic in March 2020 temporarily stopped most in-person community engagement in the building, which had been key to much early work to build a shared COS in evolv1. While efforts continue to help shape this culture, participants did acknowledge that the COVID-19 pandemic has doubtless negatively impacted the momentum of building a shared COS within the building, at least in the near-term.

#### Key takeaway and discussion: participants know much of what is required to grow a COS, but need further supports to contribute effectively to its growth at evolv1

Tellingly, participants had no shortage of ideas for what would be required to build a COS at evolv1. The rich and varied responses to Q1 paint a wide-reaching yet also precise view of what is required to support a thriving COS in the evolv1 context, resulting in the three overarching themes. While participants indicated all three themes for building a COS were present in some minor form in evolv1, all three also included areas for significant improvement to achieve a truly thriving COS in the building.

Aspects of the three themes that participants observed may be holding back the development of a COS include, among others, the perception that currently many building citizens do not seem to exemplify a strong individual interest and commitment to sustainability through their actions (indeed, some participants felt their own interest was in the minority), and that there may not yet be enough community-building for collective action on sustainability taking place at evolv1, or enough directed sustainability messaging or supporting elements of the building environment itself, to substantially increase a building-wide COS. As a result, while participants were able to describe many key ingredients required to help grow a COS, there was a general sense of frustration that many of these ingredients were only being partially met, and that barriers still existed to supporting the growth of a COS within the evolv1 context. This links to the TGB Model’s emphasis on the need for the physical context of a green building to support a range of passive to active learning and engagement opportunities on sustainability at both the individual and collective levels, which it is clear at the time of this study were only being partially and insufficiently met.

Lastly, there was also the difficult question of whether a successful COS at evolv1 must include all people who use the building, or if not, then what threshold of participation would constitute forming a successful ‘culture’. To this point, when asked to imagine what a COS would actually feel like when it was successfully established and thriving in the evolv1 space, several participants emphasized a sense of a ‘buzz’ of energy and engagement on sustainability amongst people working in the building that they would immediately feel, conveying a clear sense of community collaborating together around a shared, common purpose. At what threshold of engagement this ‘buzz’ of energy is likely to be created remains an open question, however it was clear participants did not feel this level of energy and widespread engagement on sustainability currently exists within the evolv1 space (although some participants did describe an enthusiastic energy for sustainability within their own organization). This feeling of energized, widespread engagement in sustainability amongst a community of people has been described as key to building a COS in previous studies (see for example [31, 72]), and was re-emphasized as essential by participants. Such an energized (and energizing) setting would animate all those active within it to also engage on sustainability, to the point where a thriving COS could indeed emerge and be sustained moving forward.

Tellingly, when participants were asked to imagine what a successful COS would feel like at evolv1, it was this energized feeling of *acting as a community with a common purpose*, more than anything else, that was seen to be critical. This links to Cole [5]’s insight that for a TGB to support and encourage this common purpose, it needs to “meet individual learners who are at different starting points in their understanding of environmental and architectural issues [through] a multi-pronged approach to engagement” (p. 845), and links with Dreyer et al. [14]’s core principles derived from their COS theory of change, including the need for such change efforts to be participatory, comprehensive, strategic, long-term developmental, and systems-oriented. Lastly, participants’ understanding of the multilayered nature of an effective COS aligns with the ecological systems perspective (e.g., [73], among others) and is reflected in the three Q1 themes that emerged from the data, which are similarly nested within each other and interconnected, describing different levels of impact within a system that together shape the broader culture. It also aligns with an understanding of the evolv1 building itself as being nested within a broader environment, and that “each tangible artifact in the built environment is part of the ongoing narrative of our society’s relationship to its surrounding ecology” ([36], p. 110).

### Influence of building features on sustainable values and practices

In response to Q2, *What, if any, building features positively or negatively influence the sustainable values and practices of citizens and their organizations?*, participants recognized that as a green building, many building features at evolv1 already positively encourage sustainable values and practices (Q2-T1), including, among others, the solar panels, electric vehicle (EV) chargers, secure tenant bike parking, prominent stairs and living green wall. Also, multiple participants felt the building as a whole can have a positive influence on sustainability values and practices as a cutting-edge green building, e.g.,*“It’s clear with the net positive energy that we’ve moved to a new generation of building”* (P5).

In contrast, features that were seen to discourage sustainability (Q2-T3) include, among others, the large parking lot, elevators, automatic handwash stations and paper towels in the bathrooms. Citizens discussed the reality that sustainability is not always ‘pure’ (Q2-T2), and hence some features may both promote and hinder sustainable values and practices depending on their specific use and individual perspectives. For instance, one participant observed *“The EV chargers are great, but the parking lot introduces this whole lens of cars first. For people who don’t have sustainability on their conscience it’s far too easy to say ‘Yeah, I’m definitely going to drive my car every day. There’s no reason not to’”* (P4, see Additional file 1: Appendix A, Figure A2).^1^ Participants also identified several ‘missed opportunities’ at evolv1 for further promoting sustainability (Q2-T4), in particular emphasizing the lack of direct sustainability communication and education in the building (*“There’s a missed opportunity for education on the majority of the building’s features”*, P4); lack of accessible visitor bike parking; lack of art and visible creative expression; and lack of outdoor greenery as areas of potential future action. These missed opportunities were an unexpected theme, as they refer to building features or services that are seen to be insufficient or simply not there - however it was *what was missing* in and around the building context that was often described as having among the most negative influence on the sustainable values and practices of citizens and their organizations, by not more fully and explicitly encouraging key aspects of sustainability.

#### Key takeaway and discussion: acknowledging what is working, with room for improvement

Participants were careful to acknowledge the many positive aspects of the evolv1 building that are already promoting a COS amongst building citizens – however, this acknowledgement was often accompanied by constructive critique and suggestions for areas of potential improvement.

The diversity of insights shared clearly indicate the mixed impact of evolv1 on the sustainable values and practices of citizens and their organizations. As a HPGB, evolv1 does include building features that are actively promoting sustainable values and practices amongst building citizens; however, many building features that *could* be promoting sustainability are not, largely because they are currently invisible and/or unknown to many building citizens (e.g., the cistern for rainwater harvesting; the geothermal system for building heating and cooling; the solar thermal wall for air heating). When these missed opportunities are combined with the reality that some building features are still actively discouraging sustainable behaviour, it is clear that while evolv1 has already achieved a great deal as a HPGB, the building still has significant room for improvement to support the growth of a COS within it. Participants’ reflections also link to Cole [36]’s theorizing on strategies for developing TGBs, including the need to “curate learning experiences across the building to consider the total educational experience… A deeper understanding of the physical, personal, and socio-cultural contexts can increase the chances that a TGB is a successful venue for free-choice learning” (p. 112). This is reflected further in Schiller [74]’s research on the potential of green buildings as teaching tools that foster sustainability education, and Kong et al. [75]’s conceptualization of the potential for green buildings to become ‘three-dimensional textbooks’ for environmental education. Further, the importance of considering building aesthetics was raised in various forms by participants, in particular via emphasis on the lack of art or visual creative expression in the building, and lack of greenery around evolv1. This reflects a growing recognition by researchers that the beauty of architecture and built environments can also play an integral role in their overall longevity and environmental and social sustainability (e.g., [76, 77]).

### Symbolizing sustainability

In response to Q3, *How does the building symbolically communicate to people and how do symbols in the building environment translate into citizens’ own sustainability-related values and practices?,* participants’ responses showed a sensitivity to identifying ‘degrees of sustainability symbolism’ present between different building features at evolv1. On the one hand, participants recognized that some building features clearly function as symbolic green features (Q3-T1), including in particular the prominent solar panels and living green wall. *“The solar panels are a huge symbol of sustainability and are great in providing renewable energy and making us think about where our energy comes from”* (P1). On the other hand, participants observed that many sustainable features at evolv1 remain hidden, and therefore do not operate effectively as symbols. *“Some of the more hidden sustainable features could serve as symbols, but we don’t have the education around them so right now they don’t”* (P1). The building itself was seen to function as a symbol of *“where we put our values”* (P3), however some participants found the building to appear “unassuming” as they perceived it to be highly similar in appearance to other, less sustainable office buildings in the area, and therefore also less effective in clearly communicating a value of sustainability.

For symbols to communicate effectively, participants recognized the need for features to ‘stand out’, and that more visible sustainable building features operate better as symbols and are hence better able to encourage citizens’ own sustainability-related values and practices (Q3-T2) (see Additional file 1: Appendix A, Figure A4). The relative lack of sustainable features that do ‘stand out’, aside from the solar panels, central stairs and living green wall, can be seen as another missed opportunity. By enhancing sustainable features with improved public education and communication, including hidden features, participants felt there was still potential for them to function as far more effective sustainability communicators and symbols, and better support a COS (Q3-T4). *“Education is part of the low-hanging fruit for growing a culture of sustainability. There’s a lot more that’s already in the building to be celebrated and shared to influence culture”* (P1) (see Additional file 1: Appendix A, Figure A5). Here, there is clear overlap between the perceived influence of building features on sustainable values and practices (discussed above) and their perceived effectiveness in operating as sustainability symbols, of which clearer sustainability education and communication was seen to be an important strategy for bridging the two.

Similar to a missed opportunity, the absence of something important in an environment was seen to possibly create a ‘negative symbol’ for sustainability (Q3-T3). These absences included frustration expressed at the lack of public imagery and art linked to sustainability within the building, lack of educational signage describing the building and building features, and lack of greenery around the building, among others. *“There’s nothing in the garden, which I think is a negative symbol, because we have so much greenery inside the building but so far not much really outside”* (P1) (see Additional file 1: Appendix A, Figure A6). Aside from a single information screen, one participant commented that *“there’s no explanation of what’s happening in the building, and no plaque that identifies key details about the building”* (P2). Lastly, there was recognition that symbols of sustainability may not always be sustainable, raising concerns over symbolism versus actual sustainability (Q3-T5). *“The living wall is really cool and it’s one of my favourite aspects of the building. And I think it depicts nature, but I don’t know if it depicts sustainability”* (P6).

#### Key takeaway and discussion: need to link sustainability symbolism with direct education and engagement

Cole [36] emphasizes that “one basic role of a green building is to stand as a symbol of culture change… If we understand green buildings as having an interpretable message, then we can further acknowledge that architectural “language” varies enormously across different green buildings” (p. 110–111). Connected to this, participants recognized that not all sustainable building features at evolv1 clearly communicate sustainability or a ‘sustainable ethos’ [37], which is concerning as contextual factors such as conveying a clear value of sustainability are important to encouraging individual sustainable behaviours (see for example [78]). To address this, many participants recommended: that *all* sustainable building features (both visible and non-visible) be accompanied by clear educational signage to ‘bring to life’ the educational value of that feature, and to deepen viewers’ ability to connect with each feature and understand its links to sustainability; and/or to provide opportunities for engagement to enable viewers to more easily interact with each other and the building features in question, again centering sustainability. Notably, formal educational signage is one of the key ways that occupants learn about sustainability in green buildings, and can appear in many different forms depending on design choices and physical context (e.g., [8, 79]) – further affirming participants’ recommendations for inclusion of educational signage within the evolv1 building. Participants’ observations also align with the TGB Model’s emphasis on opportunities for social interaction and physical engagement with the building space, and that “a green building can also be designed for users to actively engage with its features” ([36], p. 112) to then promote more active, hands-on, ‘situated learning’ [80] within the green building environment.

Notably, where some participants did express that specific building features were shifting their own behaviours to be more sustainable, this was often associated with the utilitarian function of those features over their symbolic value (e.g., the availability of prominent stairs in the evolv1 atrium encouraging people to take the stairs). Here, the features themselves are designed to be directly used, engaging the user; however, they are often still lacking additional clarity over what makes them sustainable which could be enhanced through signage and education. Some features with a stronger symbolic value rather than a clear sustainability function were also described by participants as having some impact on increasing sustainable behaviour (particularly the living wall), however this impact was still seen to be greatly enhanced if the sustainable elements of these features were also described through accompanying educational signage, and/or opportunities for engagement (e.g., a sustainability workshop centered around the living wall). These findings align with the TGB Model’s design pattern suggestion to communicate factual information on sustainability (verbal or image-based), for instance over architectural features [5] to help maintain a “visible culture of sustainability” (p. 850) and that “TGBs, with the intent to educate users, may benefit from architectural design that outwardly communicates green intent” ([36], p. 110), along with “strategies that are more deeply intertwined with the social dynamics of the people in a space (an educational programming approach)” (p. 113).

Participants did acknowledge and appreciate that there is already some effort toward sustainability education and engagement within evolv1. However, such education and engagement was still seen to be insufficient and somewhat exclusive to particular groups, often not engaging those who are not already part of that group and interested in sustainability. Rather than only promoting sustainability at certain times and to particular audiences, many participants imagined a building where ‘everything’ (or most things) promoted sustainability, reaching all building users. This aligns with an understanding of the need to promote more widespread ‘green building literacy’ [81] amongst all those who interact with and use the building. Direct educational signage on sustainability (passive engagement) and more opportunities for experiential engagement on sustainability (active engagement) by *all* building users are some clear pathways for moving evolv1 toward being a far more effective TGB.

In sum, while symbolic function was seen as having its own inherent value for promoting sustainability, the existing sustainability symbolism in evolv1 was not seen as sufficient on its own to promote a shared COS in the building. This reflects participants’ understanding that while symbolism matters, to be most effective it needs to be bold, ‘stand out’, and be coupled with education and engagement on making more sustainable choices as complimentary key aspects of growing a successful COS. This aligns with Cole’s [36] finding that “TGBs are designed to communicate and engage. A thoughtfully designed TGB could have symbolic importance to users while affording a variety of opportunities to learn about sustainability and make a difference through participating in pro-environmental activities” (p. 114).

### Further promoting sustainability in evolv1

In response to Q4, *What could be done to further promote sustainable values and practices at evolv1?*, participants identified many potential actions that could be taken, some of which have already been mentioned. Major recommendations included the importance of reconsidering the function of spaces within and around the evolv1 building to center sustainability and community-building (Q4-T1), including improving the function of the open meeting area on the main floor to be more of a ‘magnet’ for tenant organizations, community and sustainability events (see Additional file 1: Appendix A, Figure A7); introducing a publicly accessible café; and redesigning outdoor spaces to create more useable, attractive green gathering spaces. *“I remember in the summer we did something outside and we all just sat on the ground. There wasn’t anywhere for us to sit. So just that idea of providing space outside for people I think is really important”* (P3).

The importance of combining existing symbolic green features with direct sustainability education and engagement was also emphasized, including co-creating and promoting sustainability workshops and events, and adding sustainability signage, art and direct messaging into the evolv1 space (Q4-T2). To support the education component, many participants recommended incorporating educational signage throughout the building. Several different forms were suggested for how signs could appear, including both physical and digital options. As one participant suggested “*On the Hub screen it would talk about upcoming sustainable events or functions that we can take part in; ways we can collaborate, lead and work together; and ways that we can submit sustainable ideas to be considered”* (P2). A common message was that clear educational signage in some form would greatly increase the positive impact of existing sustainable building features into shaping a shared COS, particularly if signs were designed to relate sustainable building features to the lives and sustainability interests of participants and building citizens (for more on the potential of strategically designed signage to promote environmentally responsible behaviours, see for example [82]). Participants’ suggestions for signage included targeted messaging on how a particular feature was sustainable, as well as how lessons from a feature could be translated into viewers’ own sustainable values and practices, e.g., asking *“how can I translate that into other aspects of my life?”* (P4).

The recognition that sustainability often requires some guidance led to rich discussions on the need for targeted programming and building policies to encourage more sustainable behaviours, and discourage less sustainable (e.g., incentivizing more sustainable transport, and discouraging solo driving) (Q4-T3) (see Additional file 1: Appendix A, Figure A8). For tenant organizations, having a *“morning chat”* (P5) on sustainability to keep the conversation as part of the organizational culture was recommended. Supporting sustainability leadership and community-building were emphasized as key for growing a COS at evolv1, with participants recommending a range of potential strategies to help, most of which centered on the need for building citizens to know how they can lead change within the space, and the inspiration and empowerment to do so. Inspiration to lead change was further linked to connection to nature (see Additional file 1: Appendix A, Figure A9) and community within and around evolv1, and the feeling that this change leadership would be supported (Q4-T4). However, several participants described a feeling of an overall shortfall in support for change leadership and empowerment presently at evolv1, e.g., *“There’s probably a lot more that the building and people could do that would be empowering for everybody”* (P2). Participants also emphasized the importance of maintaining consistency in the building’s message, which can then also help building citizens maintain their own focus on sustainability, e.g., *“If you’re promoting green, try to make sure that you only provide green options”* (P1).

#### Key takeaway and discussion: need for greater empowerment to act for sustainability

The cross-cutting theme of empowerment to be able to act to help build a shared COS appeared repeatedly across many of the sixteen themes, in response to multiple questions. This included the recognition that building citizens must be empowered to participate in sustainability action within the evolv1 space, including both knowing *how* to participate (e.g., [83]) and having the freedom (within limits) to do so. This finding aligns with relational empowerment-related theory in community psychology as well (e.g., [30, 84]). Many of participants’ own recommendations for changes to the evolv1 space (section ‘*Further promoting sustainability in evolv1*’, above) were also empowerment-related, and related to an understanding of strengthening the three core dimensions of sustainability (environmental, economic, and social; see [17]).

While participants acknowledged that some empowerment to take sustainability action does exist at evolv1, this was overall seen as insufficient, and there was generally a sense of confusion over the ‘proper channels’ to go through to propose and/or initiate a potential new sustainability action. Here, participants expressed feeling unable to take action by not knowing acceptable ways to take action within the evolv1 space, creating a sense of discouragement and a clear barrier toward trying to initiate new sustainability actions, either independently or collaboratively with others. This is troubling, as research suggests that empowerment and sense of agency are both key for building a COS (e.g., [14, 16]), both of which appeared to be lacking for pursuing novel sustainability initiatives in evolv1.

In addition to aspiring to an environmental context at evolv1 that is more empowering and supportive of an emergent COS, participants recognized their own agency to act to help grow a COS within evolv1 and felt that *all* evolv1 citizens need to feel empowered to act to help grow this shared culture together. Present efforts to support the growth of a COS at evolv1 were appreciated but still perceived as insufficient to fully empower building citizens to co-create and take ownership of new sustainability initiatives that could form the foundation of a truly successful, thriving COS in the evolv1 space. A lack of dedicated, functional gathering space and resources for bringing building citizens from different tenant organizations together to collaborate on sustainability initiatives was also seen to hinder the long-term growth of a COS at evolv1. This aligns with the TGB Model’s emphasis on the importance of opportunities for physical engagement on sustainability, including “physical spaces in which… groups can self-organize for ongoing environmental action” ([5], p. 848), which was generally seen by participants to be lacking at evolv1. It also aligns with an appreciation of the differential in decision-making power between occupants in evolv1, which can further constrain occupants’ feelings of empowerment in taking sustainability action.

The influence of environmental context and differentials in power on feelings of empowerment was also recognized within the ‘sub-environments’ participants recognized as existing within evolv1, in particular between different tenant organizations. These organizational environments (both physical and sociocultural) can clearly also have an influence on individual employees’ real or perceived abilities to act on sustainability. Here, participants observed a clear relationship between an organization’s own sustainability engagement and the sustainability engagement of individuals working for these organizations. This further underscores the need to engage the management of evolv1 tenant organizations in promoting sustainability, in addition to engaging individual employees. Further, sustainability engagement across organizations can be further complicated and challenged when the business models and objectives of tenant organizations themselves are not necessarily grounded in sustainability. Here, Cole [36] emphasizes the need for ‘direct feedback’ on occupant performance, including tenant organizations, “in conjunction with other behavior change interventions (such as information campaigns, incentives, and evoking social norms)” (p. 117).

Linked to empowerment, several participants expressed frustration that automation, rather than personal agency, appeared to be a norm within the evolv1 building. In particular, the automation of the bathroom faucets and soap dispensers were frequently described as disempowering and also potentially wasteful of soap and water, leading participants to feel they lacked direct control over their own personal resource use. Importantly, whether or not more resources were indeed being used due to automation, to some participants their very lack of direct control over personal use of resources was seen to be disempowering and counter to a COS (for a study exploring ways in which automation can undermine individual sustainability action, see for example [85]). This again relates to the TGB Model’s emphasis on opportunities for physical engagement, and that the lack of ability to engage meaningfully with a physical space can create an experience of disempowerment. In contrast, as argued by Cole [36], one possible task of green buildings is to “shock and delight, decrease apathy, and re-sensitive people to the possibilities of a new relationship to nature through built form” (p. 110) – in so doing, increasing sustainability engagement and feelings of empowerment. While the atmospheric qualities of green buildings can motivate environmentally responsible behaviours, on their own they are often insufficient (e.g., [86]). To enhance these qualities, best practices for sustainability engagement in green buildings can include “informing occupants of behavioral options, persuading occupants to participate, and, on another extreme, actually determining their behavior through building design” ([36], p. 117). This further relates to Hamilton’s [37] concept of ‘*prime, permit* and *invite*’ as distinct approaches to designing characteristics of green buildings to better promote and enable building occupants’ environmentally responsible behaviours.

Notably, some of these study results may point to a tension between building sustainability and personal wellbeing (e.g., a lack of personal control over building temperatures may be good for building sustainability, but less good for personal wellbeing and empowerment), which could be further explored. This is further discussed in the wellbeing exploration of this research study, presented in Abel et al. [55]. However generally, a lack of control and influence over, or even understanding of, many of evolv1’s sustainable building features; current sustainability initiatives; or how to initiate new sustainability actions in the space, all led to an overall sense of *disempowerment* in working toward a shared COS in evolv1. In order to build a COS in evolv1, these valid concerns clearly need to be addressed.

#### Key takeaway and discussion: building a ‘Micro-movement’ for sustainability

Linking the personal with the collective was the recognition that to build a COS requires far more than individual action alone, but rather the growth of a collective effort with shared purposes for promoting sustainability engagement within a given context. Within the context of the evolv1 building this common desire, expressed by several research participants, could be understood to involve growing an engaged and inspiring ‘micro-movement’ for sustainability amongst all (or at least most) evolv1 citizens. Here, the cross-cutting theme of the need for community-building for collective action on sustainability was clear. While participants recognized that aspects of evolv1 do already promote some individual sustainable behaviours, a significant gap remained in the building promoting and enticing collective action. It seems clear that without strong supports and encouragements for broader building-wide collaboration and collective action for sustainability, an effective COS is unlikely to emerge in the evolv1 building space.

Here, research participants echo what sustainability and social science literature also reflects, that an individual’s actions are important, but not sufficient alone, to building a culture (e.g., [15]). Other structural pieces to empower collective action around shared purposes are also required. As argued by Harré et al. [16], adopting a shared, additional collective purpose – such as the purpose of centering sustainability – is key to facilitating a successful ‘phase transition’ [87] that enables deeper social systems change, such as toward a shared COS. Likewise, applying systems thinking can help changemakers to better target sustainability interventions at the most effective leverage points of a system ([88, 89] in [14]). Finally, supporting opportunities for stronger human-place bonding and human-to-human bonding within the building could help to better support the needed wellbeing [90] and social justice [18] elements of an emergent COS. In total, combining these alongside other strategies could enable evolv1 to join other TGBs in embodying a “dual role of physically conserving resources while also becoming beacons for an ethic of environmental care” ([36], p. 111) amongst building users and occupants – a role that most research participants and many buildings citizens appear to want the building to take up, if it is to more effectively promote sustainability.

### Summary of findings

Summarizing the core research findings, it is clear that evolv1 building features have a range of impacts on building users, including both promoting sustainable values, norms and practices, and in some cases (whether intentional or not) promoting unsustainable behaviours. In this sense, sustainability at evolv1 is not ‘pure’, and the building can still be seen to embody several missed opportunities to promote sustainability, including a lack of public education on the majority of the building’s features. Participants also spoke to three core overarching themes for growing a COS within evolv1, including: individual interest and commitment to sustainability; community-building for collective action on sustainability with shared purposes; and being embedded within an empowering, healthy and enabling environmental context.

Reflections on the symbolic nature of evolv1 include understanding the building itself as a symbol of ‘where we put our values’, along with recognizing the sustainability symbolism of several key building features that clearly stand out at evolv1 (e.g., the living wall and solar panels). However, participants also reflected that many of the building’s sustainable features remain hidden and lack public explanation, so they are unable to operate as effective symbols of sustainability. This links to the finding that, in some cases, what is missing or invisible in an environment can unintentionally create a ‘negative symbol’ for sustainability (e.g., the lack of greenery around the building). There is a clear need to link sustainable building features and symbolism with opportunities for more direct sustainability engagement and education. Other recommendations that emerged include reconsidering the function of spaces both within and around evolv1 to center sustainability and community-building; to encourage more sustainable behaviours and discourage less sustainable; and to increase opportunities for social connection, nature connection, community-building, and sustainability leadership throughout the building, in service of growing a broader, more engaged COS.

### Trustworthiness and transferability

The research process followed over this study aligns with Whittemore’s et al. [91] established primary criteria for validity in qualitative research, including credibility, authenticity, criticality, and integrity. These qualities were promoted via triangulation of data from multiple types of sources (e.g., [92]), member checking (e.g., [71]), and clearly defined procedures for both the group sessions and one-on-one interviews. The high level of engagement throughout both the group sessions, interviews, and follow-up survey ensured a high level of richness and depth in the data, increasing its trustworthiness. Lastly, the description shared here is thick so that readers can assess if the context is similar enough to make some or all findings transferrable in some form to their own contexts – including for informing policies, practices, and engagement on growing cultures of sustainability in other green buildings, and more broadly in other diverse social settings and physical environments.

### Contributions

This study was designed to investigate how evolv1 building citizens currently conceptualize the links between sustainability symbols, practices, values & norms in the evolv1 building – Canada’s first net-positive energy commercial multi-tenant office building, and the first to receive Zero Carbon Building-Design Certification from the Canada Green Building Council [48]. The intent of the study was to inform continued efforts in shaping a COS within this space. To this end, using the *Photovoice* method provided a valuable window into the perspectives of building citizens on how the evolv1 building, tenant organizations and citizens themselves can best nurture a COS. The use of *Photovoice* in this context is unique in that it (a) applied this method with building citizens in a HPGB, while linking this to ongoing efforts to nurture a broader COS in this space; and (b) empowered participants to document not only key sustainability features of the building, but to also reflect on how these features and the building itself influence and/or embody each of the core aspects needed for building a strong COS: values and norms, symbols, and practices.

Further, this study applied a novel adaptation of the *Photovoice* method, by mixing both participant-taken photographs with participant-selected photographs taken by researchers. This adaptation was made necessary due to restrictions on participant access to the evolv1 building at the start of the COVID-19 pandemic. In part a limitation, this adaptation can also be seen as a contribution to an evolving *Photovoice* methodology, expanding the potential range of options for future ‘hybrid’ physical-virtual *Photovoice* studies, including the potential range of people who could participate in a *Photovoice* study across broader geographies.

This study contributed to both participants, the development of the local clean economy and the broader COS literature in several ways. First, the *Photovoice* process empowered participants to meaningfully reflect on and engage with their physical environment, facilitating critical dialogue and generating collective knowledge through discussion of their own taken and selected photos. This dialogue can lead to opportunities to act on identified concerns, improving participants’ own lived experiences and sense of agency within and beyond evolv1, along with deepening a growing, shared COS within the building.

Second, as an early and pioneering local clean economy project, evolv1 is setting the tone for the emergent clean economy in its geographical area. The building itself and how culture is shaped within it has numerous implications to the future development of the clean economy across Waterloo region, including approaches taken to integrate concerns for both social and environmental sustainability, including social justice, throughout this transition. Hence it is important to know: *what is the tone that the evolv1 building is setting for guiding this sustainability transition in Waterloo region?* The present study contributes useful insights toward answering this question.

Lastly, this process provided insight into how a COS can be built within the context of a HPGB space, in an effort to address the well-known ‘performance gap’ that exists within these buildings and has been linked to a lack of COS (e.g., see [12]). This is also the first study known to the authors to use *Photovoice* to explore a COS within a HPGB, providing a unique contribution to informing future COS work. Results of this study both affirm and add further depth to existing understandings of ‘what it takes’ to grow and sustain an effective COS in a given context, including the need for sustainability values, symbols, rituals, norms and practices to all be supported for a COS to emerge (see [14, 17]). Many of participants’ insights, embodied in the 16 themes of this study, can be usefully applied in other contexts where efforts toward developing a COS are taking place, both within and beyond green buildings. These insights affirm the important and rich interconnections of ‘key ingredients’ needed to support a COS (e.g., [15]), and that to be most effective individual COS components cannot be supported piecemeal, through half-measures, or in isolation, but instead must be supported in holistic ways that recognize their interconnections. This includes, for instance, the need for individual interest and commitment to sustainability, *and* community-building for collective action on sustainability with shared purposes, *and* an empowering, healthy and enabling environmental context – which when supported together can all be mutually reinforcing, further inspiring sustainability action. Building on related literature (e.g., [14, 28]), this study furthers understanding of how specific environments – in this case, a green building – can influence and shape the development of culture generally, and the development of COS specifically, supported by a given space. Finally, this study usefully connects core attributes of recent theory on TGBs (see [36]), to core attributes theorized as necessary for developing a COS (see [14]), to the specific results of this *Photovoice* study, making the case that working towards evolving green buildings to being *TGBs* could greatly support COS efforts within diverse green building contexts worldwide, including at evolv1.

As the development and maintenance of any lasting COS is complex, this *Photovoice* research study that took place at evolv1 remains one piece of a much larger puzzle – however, by being informed by participants’ own critical reflections, captured through an in-depth and empowering qualitative research process, we hope this will make a valuable contribution towards informing locally-specific understandings of COS and driving future sustainability action and related changes. Practical research implications shared here include a wide array of takeaways to consider for use by COS practitioners and researchers; building occupants, managers, and designers; policymakers, and many others, in the urgent cause of developing shared COS.

### Limitations

Some limitations apply to the present study. The adaptation of the *Photovoice* method to be a ‘hybrid’ physical-virtual approach with less than half of all photos being taken by participants can be seen as one potential limitation. To help mitigate the impact of this adaptation, researchers decided to focus data analysis exclusively on the verbal discussions within both the group dialogues and one-on-one semi-structured interviews, and not on the visual content of the photos themselves – an approach that is common to many *Photovoice* studies.

Building a COS in evolv1 has also been made more difficult due to the COVID-19 pandemic. While all participants at the start of the study were physically working within evolv1 at least 10 hours a week, this changed abruptly midway during the study to accommodate new work-from-home orders. It is the researchers’ view that despite this shift, this did not greatly impact the study itself as participants were still able to reflect openly on the influence of evolv1 on their own sustainability values and practices, and connect for virtual sessions - however, the inability to gather in-person within the building did greatly limit the ability of building citizens to collaborate to help shape an emerging COS.

An implied assumption (and potential limitation) made in the first research question (Q1) is that participants actually *want* to create a COS, and that if they wanted to, they would like to do so within evolv1. This is an important assumption to interrogate, as there is no guarantee that either is necessarily true. That said, given participants’ own interest in voluntarily participating in a study investigating the impact of evolv1 on their own experiences of sustainability, and given ongoing COS work in the building which many participants have contributed to, it seems likely that participants are interested in contributing to growing a COS more broadly, and at least at the time of the study were also interested in growing a COS within evolv1.

This study is also limited to a relatively small sample size of six participants, despite the initial aspiration to a larger sample size of ten. This was not surprising, however, as many *Photovoice* studies often have similarly small sample sizes due to the significant time demands from participants, and does not necessarily limit the richness and variety of the data collected. However, a small sample size may limit the generalizability of the study, especially given that the participants self-selected into the study, which may be related to an interest in COS and the evolv1 building.

A final potential limitation is that half of the study participants (3 of 6) were working with SWR at the time of the study. Considering SWR is a sustainability-focused organization, this may have further biased some aspects of data collection, limiting the study’s generalizability. However, the researchers did strive to bear this potential organizational bias in mind when conducting the research, for instance by asking all participants to think more broadly about the impacts of evolv1 on building citizens generally when answering questions, in addition to perceived impacts on themselves personally. Also, the remaining three participants did work with three other organizations in the building, hence increasing overall representation to four different evolv1 tenant organizations with employees participating in the study.

## Conclusion

As articulated by Cole [36], “a green building can be a “call to action” for environmental stewardship” (p. 116). Findings from this study indicate that within the context of the evolv1 green building, participants do indeed understand what a COS means to them, as well as existing barriers and enablers within evolv1 toward achieving this. In addition, participants recognized the impact of specific green building features on their own personal values and practices as they relate to sustainability, including the influence of sustainability symbolism within the building environment. Lastly, participants articulated specific recommendations for further promoting and growing a COS at evolv1. These recommendations and reflections can help inform future COS work at evolv1, within other green buildings, and other environments where COS work is taking place. Knowledge mobilization through the implementation of three public exhibits and an article based on this study helped ensure lessons learned are shared with a broader audience, amplifying the impact of this work further.

The initial framing of this study was based on the need to develop a citizen-supported COS to help address the performance gap that is a common challenge in green buildings. This included the reflection that while the sustainable features of green buildings can sometimes contribute passively toward promoting sustainable values and practices amongst users, these features are insufficient on their own to building a COS [12]. To support the growth of a COS in evolv1, study results were further discussed in the context of Cole’s [5] Teaching Green Building Model for Learning (TGB Model). Part of the implied interest in the study was to see whether evolv1 could be said to be a TGB supporting the growth of a COS within it. To be clear, becoming a TGB is not the *only* way for evolv1 or other green buildings to support the growth of a COS within (and beyond) their walls. However, as has been argued here, there are many aspects to the TGB Model that would help to support the growth of a COS, some of which may indeed be essential to promoting this culture and linking it to the sustainable features of evolv1 as a HPGB. It is worth re-emphasizing Cole’s [36] useful characterization of TGBs as going “beyond typical green buildings to invite users to take part in the meaningful work of environmental protection” (p. 119). Given the emphasis on both sustainability communication and action in shaping an effective TGB, it is clearly important to assess the degree to which “the message of the importance of sustainability is received” ([8], p. 827), and then acted upon by those using and interacting with the building environment in assessing any potential TGB.

While the results are mixed, participants’ responses suggest that overall the evolv1 building still falls short on effectively conveying the message of sustainability to building users, either through direct sustainability messaging in public spaces or in-person sustainability education, both of which appeared to be lacking at the time of the study. While the latter has clearly been impacted by the COVID-19 pandemic making in-person education more difficult, the former was missing prior to the pandemic also. Hence, it is our view that currently, despite being a technologically impressive HPGB, evolv1 is not yet an entirely successful TGB. Recognizing ‘teaching green’ as a continuum, whether evolv1 is moving toward being *more* of a TGB remains an intriguing, open question, which all building citizens can have a continued influence on. In addition, the theory of change developed by Dreyer et al. [14] on how to create a COS in green building spaces – including with deeper considerations of equity and social justice – offers a compelling guide to further supporting the growth of this culture at evolv1, which could also intersect with and help inform future efforts toward shaping evolv1 to be a TGB. While outside the scope of this current study, further research could investigate more directly the relationship between a TGB and growing a COS in green building spaces, including how to transform a green building that is presently less adept in ‘teaching green’ into one where this skill set and embodiment is stronger. Greater emphasis on sustainability education, messaging, participatory action, social justice, direct engagement and empowerment to take action on sustainability within evolv1 would, we believe, go a long ways toward building a thriving COS within the space.

## Supplementary Information

**Additional file 1: Appendix A.** Select Photos From the *Photovoice* Study. **Figure A1.** Photo 1: Bicycle in the Parking Lot of the evolv1 Building (Illustrative of Q1-T1: Individual Interest and Commitment to Sustainability). **Figure A2.** Photo 2: Large Parking Lot at evolv1 (Illustrative of Q2-T2: Sustainability is Not Always ‘Pure’). **Figure A3.** Photo 3: Living Wall in the evolv1 Atrium (Illustrative of Q3-T1: Certain Building Features Clearly Function as Symbolic ‘Green Features’). **Figure A4.** Photo 4: Solar Panels Covering the evolv1 Parking Lot (Illustrative of Q3-T2: Symbolic Communication Often Requires ‘Standing Out’). **Figure A5.** Photo 5: The ‘Make Change’ Classroom in evolv1 (Illustrative of Q3-T4: Sustainability Communication and Education are Distinct From but Connected to Sustainability Symbolism). **Figure A6.** Photo 6: Bare Garden at evolv1 (Illustrative of Q3-T3: What is Missing or Invisible in an Environment can Unintentionally Create a ‘Negative Symbol’ for Sustainability). **Figure A7.** Photo 7: Seating Area in the Hub at evolv1 (Illustrative of Q4-T1: Reconsider the Function of Spaces Within and Around evolv1 to Center Sustainability and Community-Building). **Figure A8.** Photo 8: Cycling and Walking Trail That Connects to evolv1 (Illustrative of Q4-T3: Encourage More Sustainable Behaviours and Discourage Less Sustainable). **Figure A9.** Photo 9: Leaf Floating on Water (Illustrative of Q4-T4: Increase Opportunities for Social Connection, Nature Connection, Community-Building and Sustainability Leadership).

**Additional file 2: Appendix B.** Concise Descriptions of Themes.

## Data Availability

The datasets generated and/or analysed during the current study are not publicly available due to the personal nature of the qualitative data informing this study that could compromise research participant privacy/consent, small sample size making guaranteeing anonymity difficult if data were to be publicly released, and lack of permission secured from the research participants for public release of the data.

## Footnotes

1 Note that we as authors are aware that access to a parking lot was an area of concern particularly for employees commuting to evolv1 for work from surrounding smaller towns within and beyond Waterloo region, where reliable access to efficient public transportation remains an ongoing issue (and in some cases, is non-existent). Hence, we wish to note that in addition to making transportation decisions based on individual-level choices, there are significant systems-level changes that also need to be supported in order to enable more sustainable individual transport options. It is also notable that some tenants at evolv1 provide employees with public transport passes, and that evolv1 has a significant number of EV chargers for electric vehicles in its parking lot.
