# Supplementary material for: Co-creating cultures of sustainability and co-imagining the teaching green building: the use of a participatory Photovoice process in a HPGB context
Source: Sustain Earth. 2022 Sep 5;5(1):2. doi: 10.1186/s42055-022-00047-y (PMC9444100; doi:10.1186/s42055-022-00047-y)

**Appendix A**

**Select Photos From the *Photovoice* Study**

**Figure A1**

*Photo 1: Bicycle in the Parking Lot of the evolv1 Building (Illustrative of Q1-T1: Individual Interest and Commitment to Sustainability)*


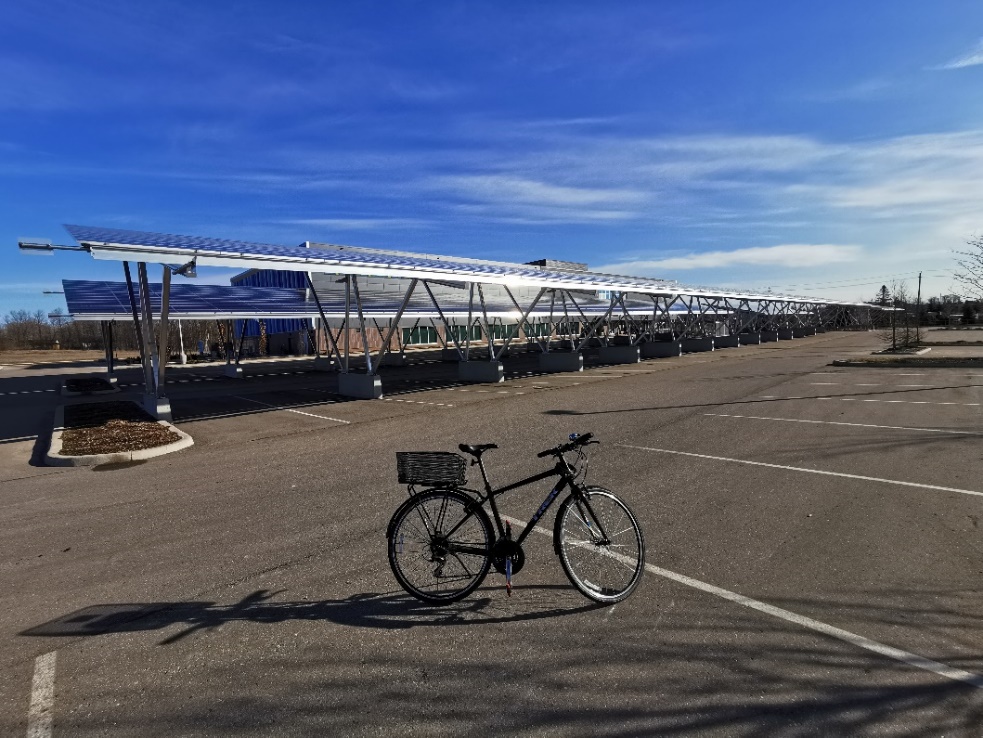


**Figure A2**

*Photo 2: Large Parking Lot at evolv1 (Illustrative of Q2-T2: Sustainability is Not Always ‘Pure’)*


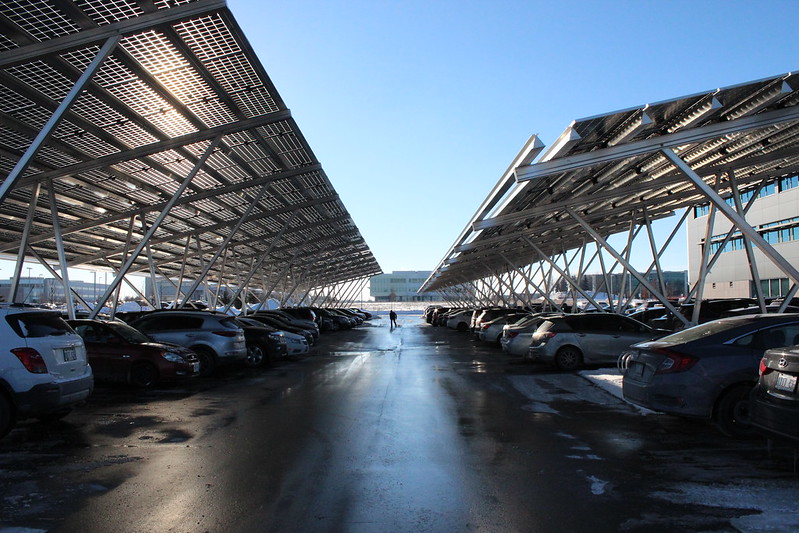


**Figure A3**

*Photo 3: Living Wall in the evolv1 Atrium (Illustrative of Q3-T1: Certain Building Features Clearly Function as Symbolic ‘Green Features’)*


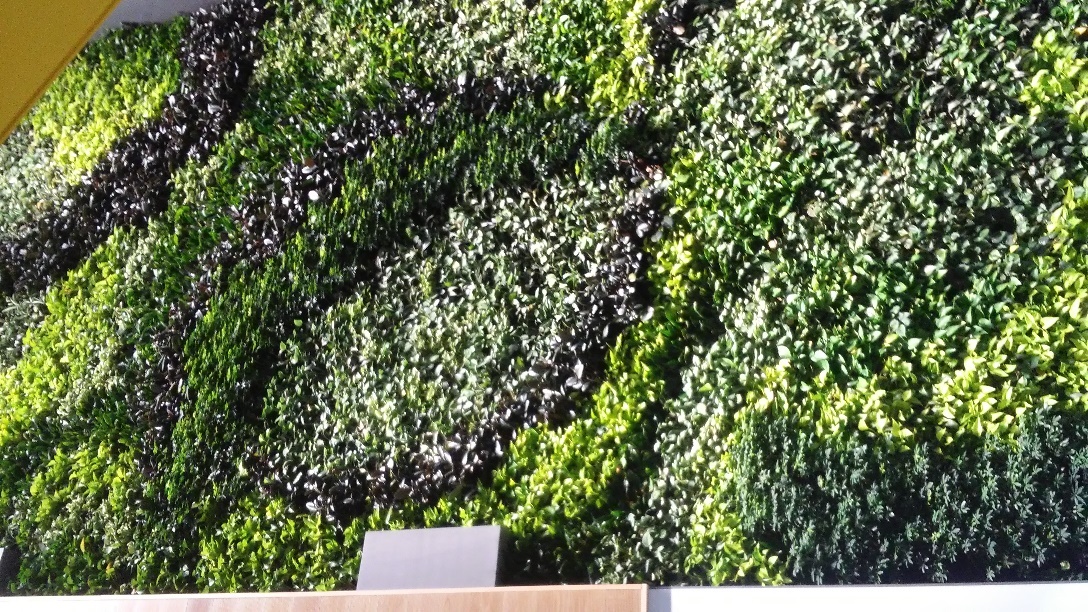


**Figure A4**

*Photo 4: Solar Panels Covering the evolv1 Parking Lot (Illustrative of Q3-T2: Symbolic Communication Often Requires ‘Standing Out’)*


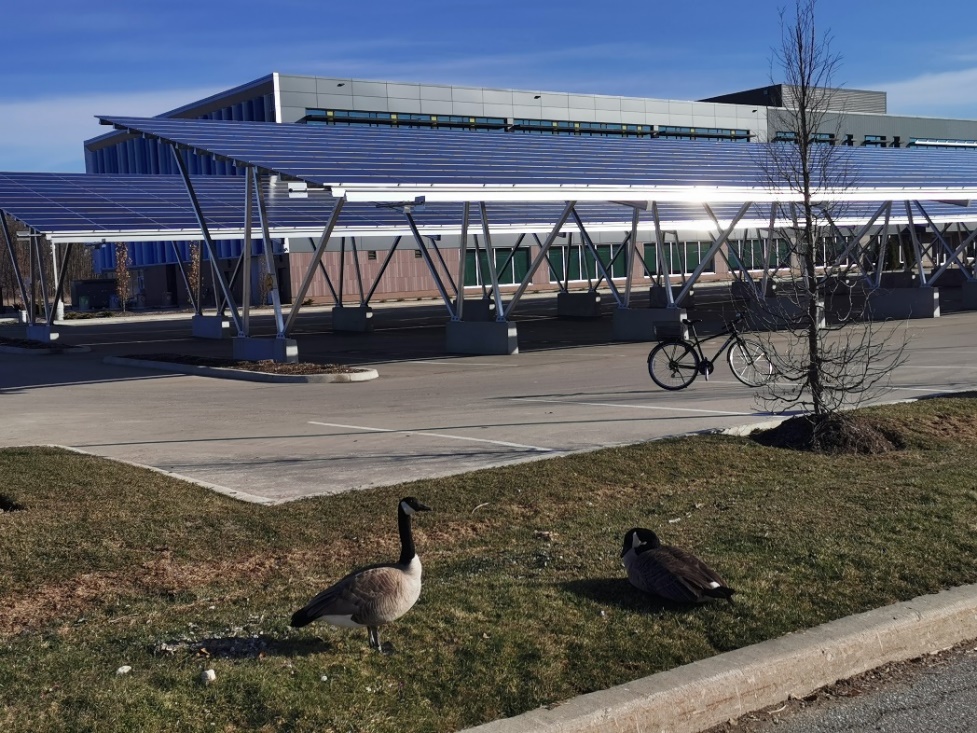


**Figure A5**

*Photo 5: The ‘Make Change’ Classroom in evolv1 (Illustrative of Q3-T4: Sustainability Communication and Education are Distinct From but Connected to Sustainability Symbolism)*


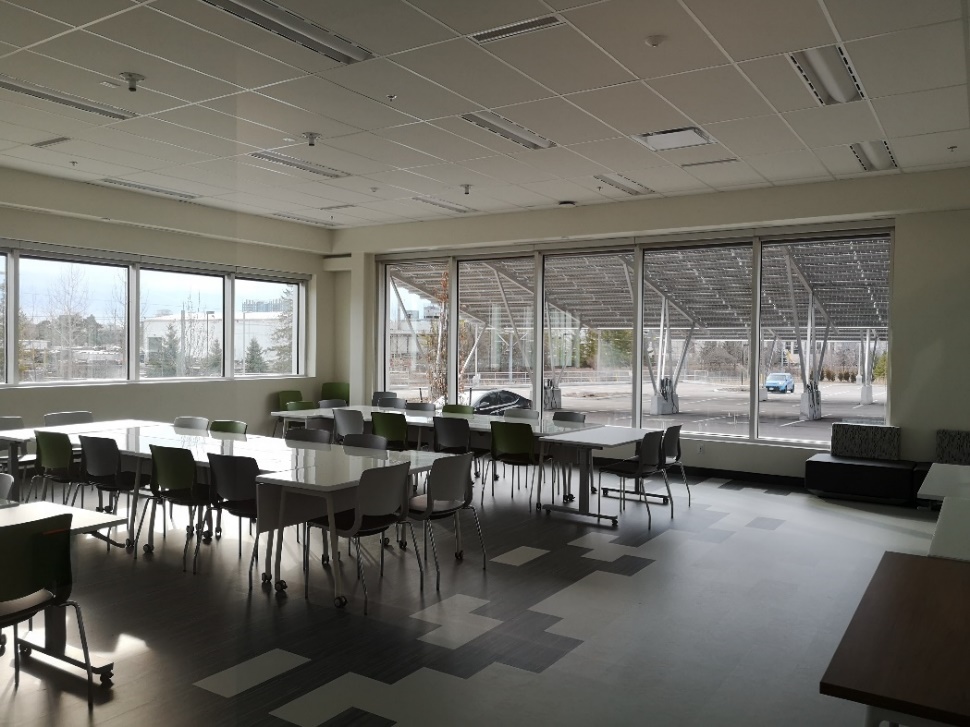


**Figure A6**

*Photo 6: Bare Garden at evolv1 (Illustrative of Q3-T3: What is Missing or Invisible in an Environment can Unintentionally Create a ‘Negative Symbol’ for Sustainability)*


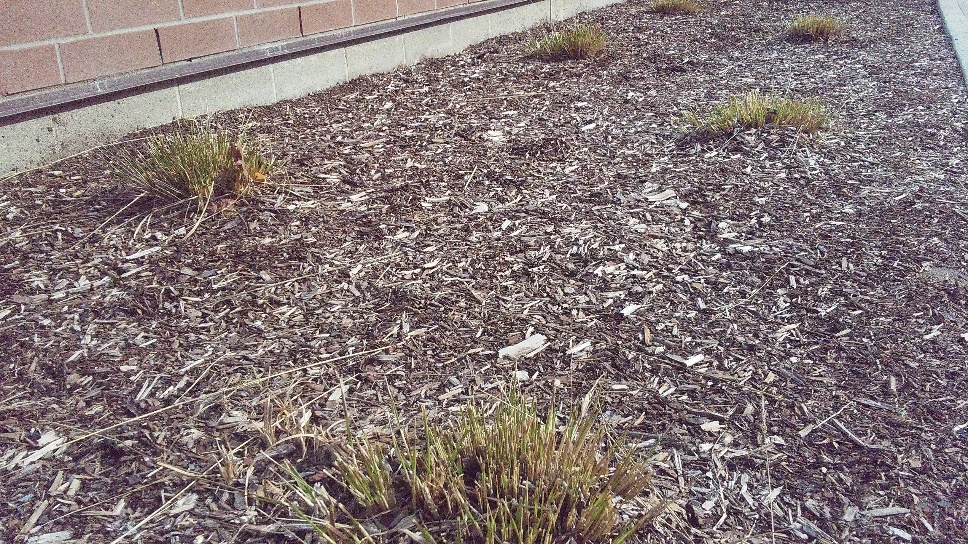


**Figure A7**

*Photo 7: Seating Area in the Hub at evolv1 (Illustrative of Q4-T1: Reconsider the Function of Spaces Within and Around evolv1 to Center Sustainability and Community-Building)*


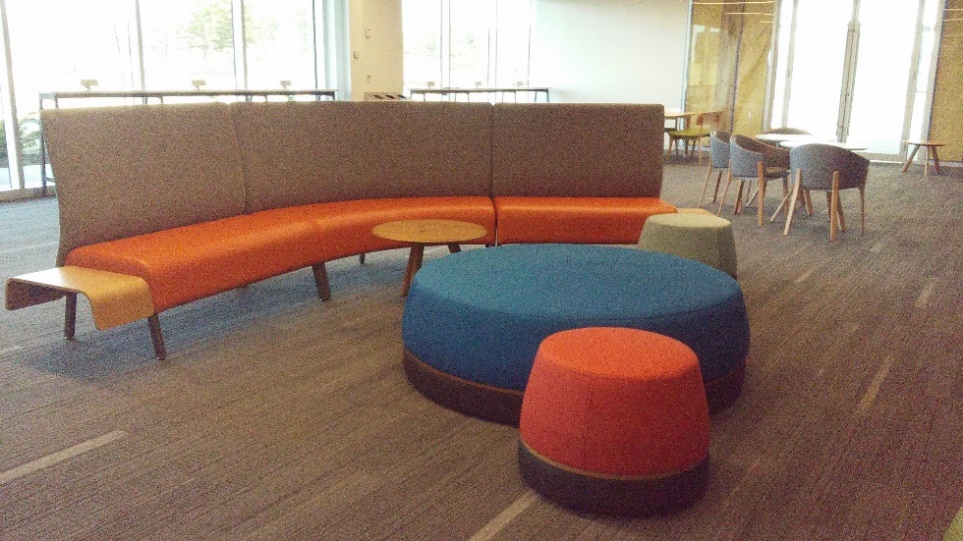


**Figure A8**

*Photo 8: Cycling and Walking Trail That Connects to evolv1 (Illustrative of Q4-T3: Encourage More Sustainable Behaviours and Discourage Less Sustainable)*


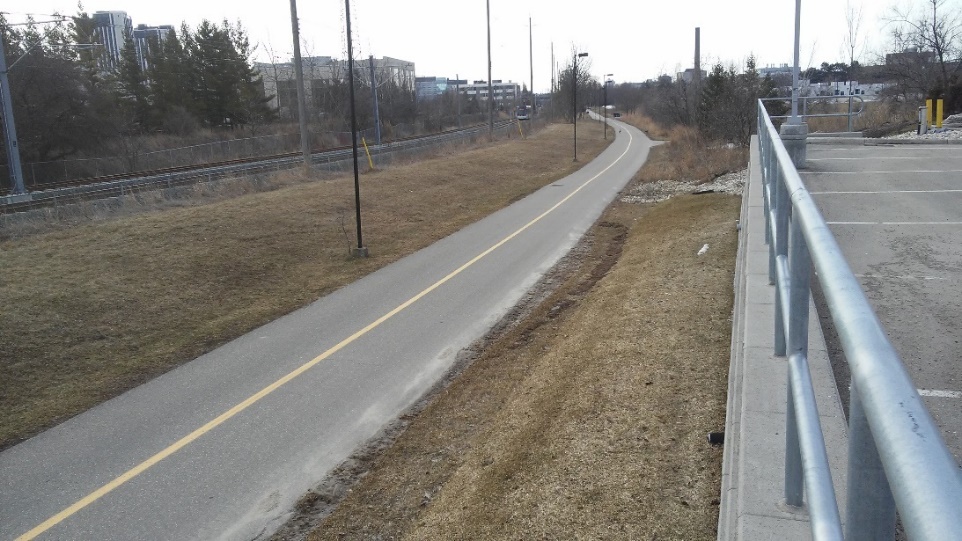


**Figure A9**

*Photo 9: Leaf Floating on Water (Illustrative of Q4-T4: Increase Opportunities for Social Connection, Nature Connection, Community-Building and Sustainability Leadership)*


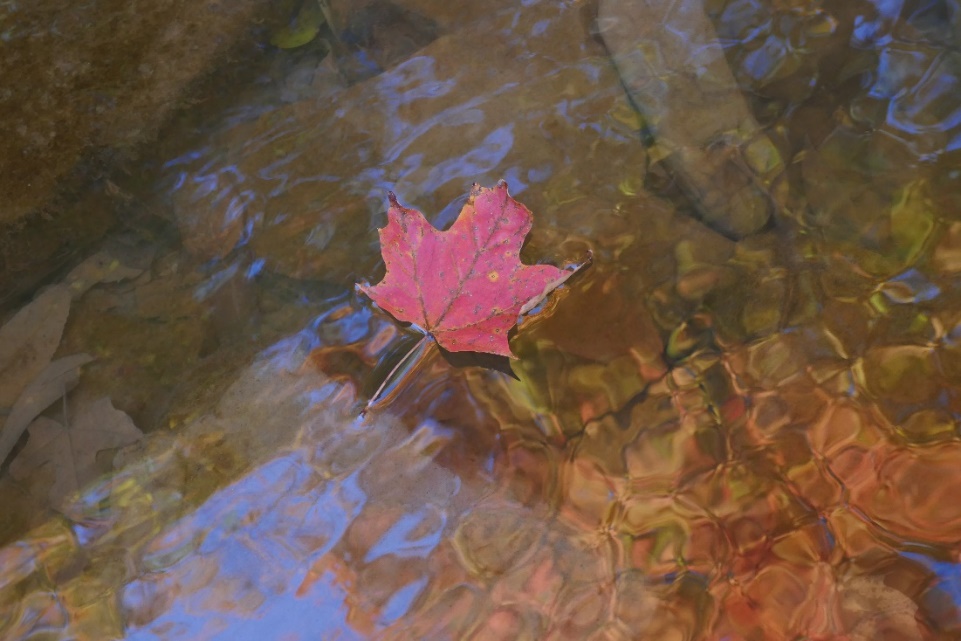

Supplement: Supplementary file 1 — Additional file 1: Appendix A. Select Photos From the Photovoice Study. Figure A1. Photo 1: Bicycle in the Parking Lot of the evolv1 Building (Illustrative of Q1-T1: Individual Interest and Commitment to Sustainability). Figure A2. Photo 2: Large Parking Lot at evolv1 (Illustrative of Q2-T2: Sustainability is Not Always ‘Pure’). Figure A3. Photo 3: Living Wall in the evolv1 Atrium (Illustrative of Q3-T1: Certain Building Features Clearly Function as Symbolic ‘Green Features’). Figure A4. Photo 4: Solar Panels Covering the evolv1 Parking Lot (Illustrative of Q3-T2: Symbolic Communication Often Requires ‘Standing Out’). Figure A5. Photo 5: The ‘Make Change’ Classroom in evolv1 (Illustrative of Q3-T4: Sustainability Communication and Education are Distinct From but Connected to Sustainability Symbolism). Figure A6. Photo 6: Bare Garden at evolv1 (Illustrative of Q3-T3: What is Missing or Invisible in an Environment can Unintentionally Create a ‘Negative Symbol’ for Sustainability). Figure A7. Photo 7: Seating Area in the Hub at evolv1 (Illustrative of Q4-T1: Reconsider the Function of Spaces Within and Around evolv1 to Center Sustainability and Community-Building). Figure A8. Photo 8: Cycling and Walking Trail That Connects to evolv1 (Illustrative of Q4-T3: Encourage More Sustainable Behaviours and Discourage Less Sustainable). Figure A9. Photo 9: Leaf Floating on Water (Illustrative of Q4-T4: Increase Opportunities for Social Connection, Nature Connection, Community-Building and Sustainability Leadership). [file 42055_2022_47_MOESM1_ESM.docx]
