# Supplementary material for: Co-creating cultures of sustainability and co-imagining the teaching green building: the use of a participatory Photovoice process in a HPGB context
Source: Sustain Earth. 2022 Sep 5;5(1):2. doi: 10.1186/s42055-022-00047-y (PMC9444100; doi:10.1186/s42055-022-00047-y)
Supplement: Supplementary file 2 — Additional file 2: Appendix B. Concise Descriptions of Themes. [file 42055_2022_47_MOESM2_ESM.docx]

**Appendix B**

**Concise Descriptions of Themes**

**Q1: What does a culture of sustainability mean for citizens of the building, and what can influence its development?**

Q1, Theme 1: Individual interest and commitment to sustainability

Description: *Some level of interest and commitment to sustainability is needed by individuals to build a culture of sustainability.*

Q1, Theme 2: Community-building for collective action with shared purpose(s)

Description: *A culture of sustainability does require attention to community-building for taking collective action with shared purpose(s), enabling advancing sustainability in particular areas.*

Q1, Theme 3: An empowering, healthy and enabling environmental context

Description: *A culture of sustainability both requires, benefits from, and helps create a broader environmental context that is empowering, healthy and enables participants to pursue sustainability actions.*

**Q2: What, if any, building features positively or negatively influence the sustainable values and practices of citizens and their organizations?**

Q2, Theme 1: Many evolv1 features already promote sustainable values, norms and practices

Description: *There are many features both in the evolv1 building itself and the surrounding evolv1 property that already promote sustainable values, norms and practices.*

Q2, Theme 2: Sustainability is not always ‘pure’

Description: *Building features do not necessarily purely promote sustainability – some building features may both promote and hinder sustainable values, norms and practices.*

Q2, Theme 3: Some evolv1 features are actively discouraging more sustainable values, norms and practices

Description: *There are some features both in the evolv1 building itself and the surrounding evolv1 property that actively discourage more sustainable values, norms and practices.*

Q2, Theme 4: evolv1 still embodies several ‘missed opportunities’

Description: *There are some building features that could better promote sustainability, but don’t, and hence can be seen as ‘missed opportunities’ (e.g., lack of sustainability signage).*

**Q3: How does the building symbolically communicate to people and how do symbols in the building environment translate into citizens’ own sustainability-related values and practices?**

Q3, Theme 1: Certain building features clearly function as symbolic ‘green features’

Description: *There are certain building features that clearly take on a role or function as a symbolic ‘green feature’ (e.g., the solar PV, and living wall).*

Q3, Theme 2: Symbolic communication often requires ‘standing out’

Description: *For a feature to symbolically communicate, it often needs to be easily noticed or seen, and hence to ‘stand out’ (e.g., the solar PV, staircase, and living wall).*

Q3, Theme 3: What is missing or invisible in an environment can unintentionally create a ‘negative symbol’ for sustainability

Description: *What is not present or cannot be seen in an environment can unintentionally operate as a ‘negative symbol’ for sustainability (e.g., lack of clear visitor bike parking; lack of greenery around evolv1)*

Q3, Theme 4: Sustainability communication and education are distinct from but connected to sustainability symbolism

Description: *While sustainability communication and education are not the same as sustainability symbolism, they are connected and can often strengthen each other (e.g., including educational signage alongside a symbol of sustainability)*

Q3, Theme 5: Concern over symbolic representation of sustainability, versus actual sustainability

Description: *While some features may clearly symbolize sustainability, that does not guarantee that they are actually fully sustainable – raising concerns over representation of sustainability versus actual sustainability.*

**Q4: What could be done to further promote sustainable values and practices at evolv1? ****Note. For Q4, the theme titles are also seen to provide sufficient descriptions; hence, separate descriptions are not included.*

Q4, Theme 1: Reconsider the function of spaces within and around evolv1 to center sustainability and community-building

Q4, Theme 2: Combine existing symbolic communication with direct sustainability education and engagement

Q4, Theme 3: Encourage more sustainable behaviours and discourage less sustainable

Q4, Theme 4: Increase opportunities for social connection, nature connection, community-building and sustainability leadership
